# Systematic site-directed mutagenesis of the *Helicobacter pylori* CagL protein of the Cag type IV secretion system identifies novel functional domains

**DOI:** 10.1038/srep38101

**Published:** 2016-12-06

**Authors:** Tobias Bönig, Patrick Olbermann, Simon H. Bats, Wolfgang Fischer, Christine Josenhans

**Affiliations:** 1 Medizinische Hochschule Hannover, Institute for Medical Microbiology, Carl-Neuberg-Strasse 1, 30625 Hannover, Germany; 2 DZIF German Center for Infection Research, partner site Hannover-Braunschweig, Carl Neuberg Str 1, 30625 Hannover, Germany; 3 Ludwig Maximilians University Munich, Max-von-Pettenkofer Institute, Munich, Germany; 4 DZIF German Center for Infection Research, partner site Munich, Pettenkoferstr. 9, 80336 Munich, Germany

## Abstract

The Cag Type IV secretion system, which contributes to inflammation and cancerogenesis during chronic infection, is one of the major virulence factors of the bacterial gastric pathogen *Helicobacter pylori*. We have generated and characterized a series of non-marked site-directed chromosomal mutants in *H. pylori* to define domains of unknown function of the essential tip protein CagL of the Cag secretion system. Characterizing the CagL mutants, we determined that their function to activate cells and transport the effector CagA was reduced to different extents. We identified three novel regions of the CagL protein, involved in its structural integrity, its possible interaction with the CagPAI T4SS pilus protein CagI, and in its binding to integrins and other host cell ligands. In particular two novel variable CagL motifs were involved in integrin binding, TSPSA, and TASLI, which is located opposite of its integrin binding motif RGD. We thereby defined functionally important subdomains within the CagL structure, which can be used to clarify CagL contributions in the context of other CagPAI proteins or for inhibition of the CagT4SS. This structure-function correlation of CagL domains can also be instructive for the functional characterization of other potential VirB5 orthologs whose structure is not yet known.

The human pathogen *H. pylori* colonizes the stomach mucosa of approximately half the world’s population. In most cases, infection causes chronic active gastritis but remains largely asymptomatic^1^. However, the course of the disease can also be associated with symptomatic gastritis and the development of gastric and duodenal ulcers^2^ and even more severe alterations of the gastric mucosa including malignant host cell transformations such as mucosa-associated lymphoid tissue (MALT) lymphoma and gastric adenocarcinoma^3^. The genome of the higher pathogenic *H. pylori* strains contains a 37 kb genomic island, the *cytotoxin associated gene* pathogenicity island (*cag*PAI) that plays a crucial role in the outcome of the infection^4^,^5^. A subset of genes in this genetically highly variable region encodes a type IV secretion system (T4SS), the CagT4SS, which induces the secretion of proinflammatory chemotactic cytokines such as interleukin-8 (IL-8) from gastric epithelial cells^6^,^7^. The expression of a functional CagT4SS mediates a profound proinflammatory response *in vivo* and *in vitro* and is thus considered one of the major virulence and cancerogenic factors of *H. pylori*. During infection, the pathogen uses the CagT4SS to transport the *cag*PAI-encoded effector protein cytotoxin-associated gene A (CagA) into human host cells^8^. Upon delivery into the target cell cytosol, CagA becomes sequentially tyrosine-phosphorylated at its C-terminal EPIYA motifs by different tyrosine kinases and influences cell functions^9^,^10^,^11^. Several CagA-dependent effects on cells and interaction partners have been identified^12^,^13^. The presence of the *cag*PAI and CagA correlates with an increased risk of cancer development in humans^14^. Studies using transgenic expression of CagA in mice^15^ and zebrafish^16^ earmarked the protein as a bacterial oncoprotein.

The *cag*PAI-encoded CagT4SS is a multisubunit protein structure that is ancestrally related to the bacterial conjugation machinery^17^,^18^. The *H. pylori cag*PAI encodes homologs of most prototypical VirB proteins, including a surface-exposed component CagC, discussed as a remote ortholog of the *Agrobacterium tumefaciens* major pilus subunit VirB2^19^,^20^, the potential VirB5 homolog CagL^21^, and additional Cag proteins that are essential for proper T4SS assembly and function^5^,^18^,^22^,^23^,^24^,^25^.

As part of a systematic mutagenesis of the genes of the *H. pylori cag*PAI, CagL was shown to be essential for CagA translocation into host cells and the induction of interleukin-8^22^. CagL exhibits features similar to other VirB5 orthologs such as its predicted N-terminal signal peptide, relatively short protein length and its hydrophobic profile which shows two hydrophobic peaks at the N- and C-termini, reviewed in^21^ and previously defined as characteristics of the remote VirB5 ortholog TraC^26^. Furthermore, CagL was localized at the tip of the surface-exposed CagT4SS structure and reported to bind to the β subunit of integrins, an important proposed host cell receptor for CagL^21^,^27^,^28^. The RGD motif within CagL can serve as a binding sequence for different integrins^27^,^29^,^30^,^31^,^32^. The important role of β_1_ integrin for CagA translocation was again highlighted independently, and three additional *cag*PAI-encoded proteins (CagA, CagY and CagI) were identified as putative β_1_ integrin interaction partners^33^. We and others have previously found that CagL is subject to positive or diversifying selection in some of its protein segments^5^,^34^,^35^. Variable amino acids in the N-terminal part of CagL have been postulated to be involved in cancerogenesis^35^,^36^. These signatures of diversifying selection may be due to different evolutionary selection pressures in the human host, such as antibody-derived immune pressure or interactions with variable host individuals and host cell receptors.

The central role of CagL in the *H. pylori* CagT4SS motivated us to perform detailed investigations to identify novel CagL segments that are crucial for the proper functioning of the CagT4SS. On the basis of previous results, we hypothesized that predicted loop regions in CagL are likely to harbor novel functional motifs. We also hypothesized that integrin interaction may not be the only mechanism mediating CagL cell interaction and CagT4SS-related functions. In this study, we have therefore characterized CagL using a comprehensive site-directed mutagenesis strategy introducing short deletions into the protein. One objective was to identify amino acid motifs in CagL that could contribute to CagL cell interaction, in an either integrin-dependent or integrin-independent manner.

Deletions in several regions of CagL influenced the amounts and subcellular localization of CagL and the activity of *H. pylori* on human gastric epithelial cells. It was possible to distinguish subsets of mutants which underwent a complete loss of CagT4SS function, mutants with intermediate phenotypes, and mutants with minor functional deficiencies. Taken together, using functional assays, we were able to identify motifs involved in the stability and subcellular bacterial transport of CagL, motifs likely involved in the interaction with other CagT4SS proteins such as CagI, and a subset of mutants in motifs other than RGD which had moderate functional deficits but seem to be involved in the interaction of CagL with integrins or other cellular components.

## Results

### CagL contains conserved and variable sequence motifs in predicted loop segments

**Figure 1:**
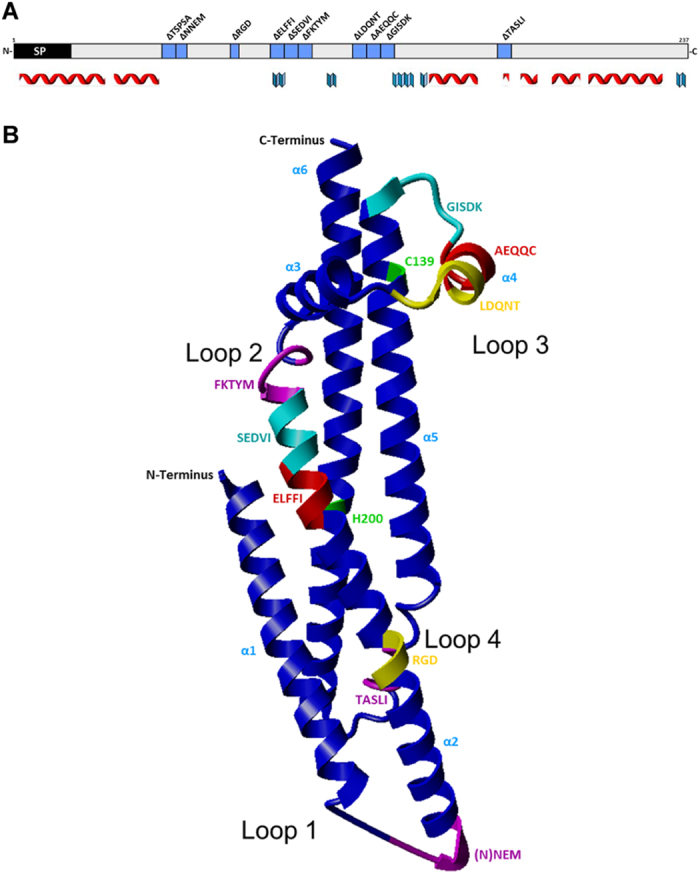
Secondary and tertiary structural prediction of *H. pylori* CagL (strain 26695); the selection of ten short motifs for generating deletion mutants is indicated with regard to the recently solved CagL crystal structure. (**A**) Placement of the ten motif deletion mutants within the secondary structure of CagL. Secondary structure prediction was performed using Jpred (www.compbio.dundee.ac.uk/jpred). Alpha helices are indicated by red symbols, beta sheets are indicated by blue symbols. Only the structured regions of confidence score ≥1 of the Jpred prediction are indicated by symbols below the sequence. SP designates a predicted leader peptide which probably serves to transport CagL to the periplasm. (**B**) Placement of the ten motif deletion mutants in the CagL crystal structure (pdb: 3zcj_chainA^38^), which was imaged in ribbon mode by Yasara (www.yasara.org). Motif deletion mutants along the 2D and 3D structure are indicated in blue (2D) and in different colors (3D structure; pink, turqoise, red, purple, yellow) contrasting to the backbone shown in royal blue. The probably disordered regions (“loops”) within CagL which correspond to predicted loop regions in a CagL structural prediction (not shown) are indicated and numbered as loop 1 through loop 4 in B (also indicated as loops with the same numbering in Supplementary Fig. S2). The TSPSA motif was not structurally resolved in the crystal (3D) structure pdb: 3zcj_chainA and therefore is not indicated in B (loop 1).

CagL, the gene product of HP0539/*cagL*, is a *cag*PAI-encoded surface-associated protein^27^ involved in the induction of IL-8 secretion and CagA translocation^22^. In previous studies, we and others have identified the HP0539/*cagL* gene as one of several *cag*PAI genes to show a signature of diversifying selection, at about 8.9% of its codons (21 of 237 amino acids (aa))^5^,^35^. The CagL structure is composed of largely alpha-helical regions interspersed by potentially exposed loops. We have now combined *in silico* prediction of secondary and tertiary structure with the recently published crystal structure^37^ in order to identify loop regions in CagL and match them with diverse or conserved protein segments in different world-wide *H. pylori* CagL protein variants. Comparing 38 CagL sequences from different globally collected *H. pylori* strains (Supplementary Fig. S1), we identified mainly three regions, aa 26 to 35, aa 55 to 62 (loop 1), and aa 170 to 175 (loop 4), which showed a larger sequence diversity between strains (for details of evolutionary diversifying selection of single amino acids, see also^5^). We found that most CagL variable regions were located within disordered or loop segments that connect the alpha-helical core (Fig. 1A,B)^38^. The prediction that some regions in CagL that are subject to diversifying selection *in vivo*^5^ might be surface-exposed is also in line with the major surface-exposed segments identified in the CagL structure^38^. Several other segments of the protein were rather conserved in all CagL variants, for instance within loop regions 2 and 3 (Fig. 1B). We have, in addition, compared CagL with various representative bacterial VirB5 proteins, of which CagL is a potential ortholog, using multiple sequence alignments (Supplementary Fig. S2; Supplementary Methods). Although the amino acid identity is low between these potential orthologs (between 15.5% and 28.8%), this approach also identified disordered segments which appear to be unique for CagL (Supplementary Fig. S1; Supplementary Fig. S2; Supplementary Fig. S3).

### Comprehensive site-directed mutagenesis of *H. pylori* CagL in its predicted loop regions

Based on the above-mentioned multiple sequence alignments (Supplementary Fig. S1; Supplementary Fig. S2), and the CagL crystal structure^38^ (Fig. 1B), we selected ten short CagL segments, mainly localized in predicted flexible protein loops, for site-directed mutagenesis (SDM), in order to delete short motifs of three or five amino acids. We anticipated that this approach should facilitate elucidating the role of these regions for the assembly of the CagT4SS, as well as for CagL integrin and possibly additional host cell interactions, without being overly disruptive to protein structure. The CagL motifs chosen for SDM included the motifs TSPSA, NNEM, RGD, ELFFI, SEDVI, FKTYM, LDQNT, AEQQC, GISDK, and TASLI (Fig. 1A,B). Five of these CagL motifs, TSPSA ELFFI, SEDVI, FKTYM and GISDK, are rather conserved in CagL of all analyzed *H. pylori* strains, and, like the RGD motif, are not present in other bacterial VirB5 proteins. A larger extent of *H. pylori* inter-strain variability was found within the selected CagL segments that are located inside longer loops or disordered regions (loops 1 and 4; NNEM, LDQNT, AEQQC and TASLI). The SDM was performed by unmarked homologous recombination of altered *cagL* alleles from a suicide plasmid into the *H. pylori* chromosome (Methods) in the highly DNA-uptake-competent strain SU2^5^. As an additional control strain, we also constructed a *cagL* insertion/inactivation mutant in *H. pylori* (SU2) by allelic exchange mutagenesis, which reproduced the results presented by Fischer *et al*.^22^. The mutants were first tested for bacterial growth, in order to provide a basis for further investigations. All mutants showed no growth defects in comparison to the *H. pylori* SU2 wild type reference strain (data not shown). We also performed template-based modelling of the predicted protein structures for the complete set of deletion mutants using the first published CagL crystal structure as template^38^, in order to anticipate severe defects of the short deletions on protein folding or tertiary structure (Supplementary Fig. S4). Some of the short deletions were predicted to exert a minor influence on protein fold, for example the TSPSA deletion, which affected the tilt of adjacent alpha helices; other deletions were not predicted to influence protein structure (Supplementary Fig. S4, see also Discussion).

### Differential expression and localization of CagL in chromosomal mutants expressing variant CagL proteins

**Figure 2:**
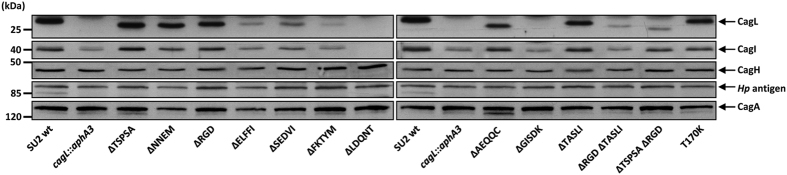
Quantification of CagL and other Cag proteins in whole cell lysates of several isogenic chromosomal CagL motif deletion mutants (strain SU2). Bacteria were lysed by sonication (whole cell lysates). Equal protein amounts of each sample (10 μg) were separated on 11.5% SDS gels followed by Western blotting. Proteins CagL, CagI, CagH, *Hp* antigen, CagA were detected using specific antisera (rabbit α-CagL, 1:20,000; rabbit α-CagI, 1:5,000; rabbit α-CagH, 1:5,000; rabbit α-*H. pylori*, DAKO, 1:2,500; rabbit α-*Hp*-CagA, 1:10,000), and matching secondary antisera. CagL, CagI, CagH and CagA amounts in each strain were quantitated by densitometry using ImageJ^56^ (see also Supplementary Figure S7). Invariable *Hp* antigen bands were used for normalization.

We then further characterized the generated set of isogenic chromosomal unmarked mutants expressing variant CagL proteins. Initially, we tested CagL expression (Fig. 2), in order to lay the groundwork for assessing functional deficits of the CagT4SS associated with the short deletion mutants.

Some of the motif deletion mutants generated by site-directed mutagenesis showed striking differences in CagL expression levels. In particular, five mutants (ΔELFFI, ΔSEDVI, ΔFKTYM, ΔLDQNT, ΔGISDK) stood out by a strong reduction in overall CagL (Fig. 2). The most substantial reduction was observed in the latter two mutants. Interestingly, the decrease of CagL in these mutants correlated with a strongly diminished detection of CagI, but no reduction of CagH (Fig. 2; see below). In four of the motif deletion mutants, CagI was surprisingly reduced to similar levels as in a *cagL* knock-out mutant, which we included as a negative control (Fig. 2). CagI was even no longer detectable at all in whole cell lysates of the ΔLDQNT motif mutant (Fig. 2), in contrast to the *cagL* knock-out mutant, which still had detectable levels of CagI. Three deletion mutants, ΔRGD, ΔAEQQC, and ΔTASLI, displayed intermediately reduced CagL amounts by about 40% to 50% (Fig. 2).

For detecting the subcellular localization of wild type and mutant CagL proteins, *H. pylori* bacteria were separated into outer (O), soluble (S) and insoluble (I) fractions (Methods). Representative results of Western blot analyses are depicted in Supplementary Fig. S5 (one of two tested mutant clones with equivalent results are shown for each deletion). In bacterial cell fractions of the *H. pylori* SU2 wild type, we detected CagL in all three fractions. The largest amounts of CagL were localized in the insoluble fractions of wild type bacteria (predicted to be enriched in membrane-associated proteins). No CagL was detected in the isogenic *cagL* insertion mutant (SU2 *cagL*::*aphA3*). The comparative analyses of CagL localization in bacterial fractions between the various chromosomal deletion mutants also revealed differences in CagL localization (Supplementary Fig. S5; Table 1). Interestingly, in one of the five mutants which showed a strong overall CagL reduction in lysates, SU2 CagL^ΔLDQNT^, which is located in loop 3 (Fig. 1B), CagL was, in addition, not detected anymore in the soluble nor in the outer fractions (Supplementary Fig. S5). In the mutant deleted for motif ELFFI, a motif which is located within the hydrophobic core of the CagL structure (Fig. 1B)^38^, small amounts of CagL were still detectable in all fractions (Supplementary Fig. S5), indicating that the membrane transport of CagL is not diminished in this mutant despite its reduced CagL amounts. Three of the five strongly expression-deficient mutant strains, SU2 CagL^ΔSEDVI^, SU2 CagL^ΔFKTYM^ at the edge of the α2 helix, and SU2 CagL^ΔGISDK^ in loop 3, exhibited a detectably reduced CagL expression in all three fractions, although they produced more CagL than the latter two mutants. The fractionation pattern for SU2 CagL^ΔGISDK^ (loop 3) also indicated a CagL transport defect (Supplementary Fig. S5), since this mutant was the only one in which CagL was still detectable in the insoluble fraction but not in the outer fraction. In two chromosomal deletion mutants, SU2 CagL^ΔTSPSA^ and SU2 CagL^ΔNNEM^, we did not note any considerable changes in CagL amounts and CagL distribution in the three analyzed fractions compared to the parental strain (Table 1; Supplementary Fig. S5). SU2 CagL^ΔRGD^ and CagL^ΔTASLI^ mutants showed slight changes in all fractions. Approaches to directly detect CagL on the surface of intact bacteria in microscopy using antibodies, which we carried out in order to refine our localization analyses in the different mutants, were not successful (data not shown).

### *H. pylori* CagL deletion mutant strains exhibited distinct differences in the activation of human gastric epithelial cells and in CagA translocation

**Figure 3:**
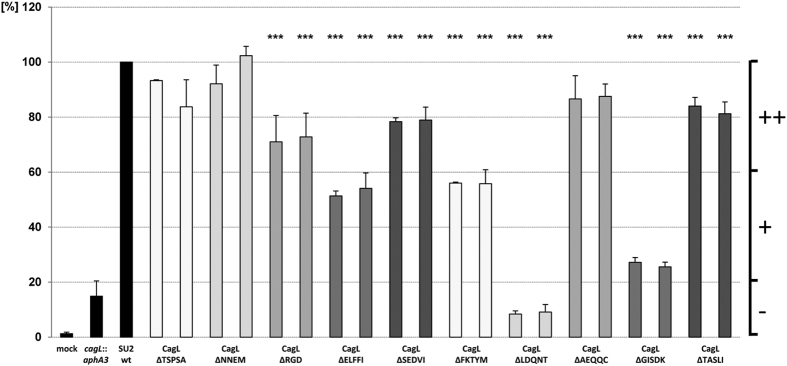
IL-8 induction capacity of site-directed short deletion mutants in CagL in comparison to the parental strain *H. pylori* SU2. AGS cells were infected for 22 h (MOI = 50) with several isogenic chromosomal HP0539/*cagL* mutants as indicated in the figure. Culture supernatants of coincubated AGS cells were tested for IL-8 by ELISA. The level of IL-8 secretion induced by contact with the SU2 reference strain was set to 100%. The corresponding values of the HP0539/*cagL* motif mutants are depicted in percent in relation to wild-type levels. Results from two independently generated and validated clones are depicted for each motif deletion mutant. The data shown are results (mean and SD) from triplicate measurements of at least four independent experiments. Significance of differences in comparison to the parental strain was calculated by student’s *t*-test shown as black asterisks above each bar: ***p < 0.01; **p < 0.02; *p < 0.05. The plus and minus signs at the right side of the figure indicate: [++] normal to slight reduction, [+] clear reduction, and [−] strong reduction.

**Figure 4:**
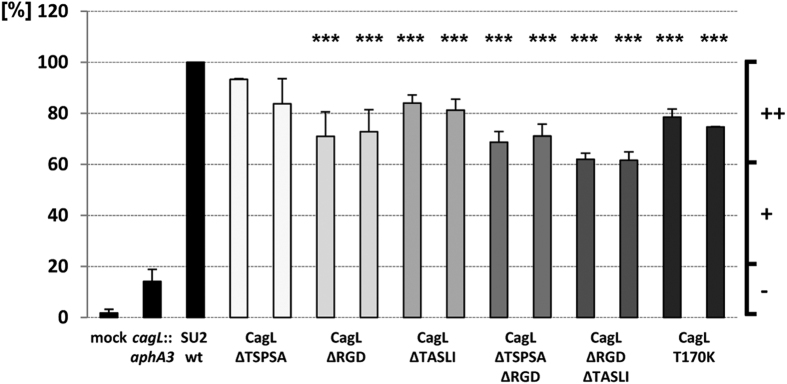
IL-8 induction capacity of selected site-directed double motif deletion mutants and single amino acid substitutions in CagL in comparison to the parental strain *H. pylori* SU2. AGS cells were infected for 22 h (MOI = 50) with isogenic chromosomal HP0539/*cagL* mutants as indicated. Culture supernatants of coincubated AGS cells were tested for IL-8 by ELISA. The level of IL-8 secretion induced by contact with the SU2 parental strain (wt) was set to 100%, and corresponding percent values of the *cagL* motif mutants are depicted. For each mutant, results were obtained from two independently generated and validated clones. The data shown are results (mean and SD) from triplicate measurements of at least four independent experiments. Significance of differences towards the parental strain was calculated by student’s *t*-test shown as black asterisks above each bar: ***p < 0.01; **p < 0.02; *p < 0.05. Plus and minus signs at the right side of the figure indicate normal to slight reduction [++], clear reduction [+], and strong reduction [−].

During the infection, *cag*PAI-positive *H. pylori* strains induce the secretion of IL-8^39^ and translocate CagA into host cells^8^,^12^,^40^. For further characterization of the comprehensive set of deletion mutant strains expressing different CagL variants, we analyzed these properties of all mutant bacteria in cell coincubation experiments (AGS gastric epithelial cells) and CagA translocation assays, in order to reveal functional deficiencies of the CagT4SS in the respective mutants. While the cells secreted IL-8 into the supernatant in response to coculture with *H. pylori* wild type (Fig. 3; Table 1), all tested motif mutants exhibited reductions in IL-8 induction in comparison to the parental strain. Similar phenotypes were shown for two separate clones each of every CagL short deletion mutant (Fig. 3). The CagL-negative control mutant (SU2 *cagL*::*km*) led to a strongly reduced IL-8 release, below 20% of the wild type, in confirmation of previous data^22^. Mutant strains SU2 CagL^ΔNNEM^, SU2 CagL^ΔSEDVI^, SU2 CagL^ΔAEQQC^, SU2 CagL^ΔTASLI^ reached values of approximately 80% of the wild type strain, reflecting moderate but significant impairments in the induction of cellular IL-8 (Fig. 3). Mutant strains SU2 CagL^ΔELFFI^ and, in particular, SU2 CagL^ΔFKTYM^, which possessed an overall strongly reduced amount of CagL in all cellular fractions, displayed an intermediate phenotype with significantly reduced values between 50% and 60% of wild type IL-8 values. The two mutants SU2 CagL^ΔLDQNT^ and SU2 CagL^ΔGISDK^, which both showed strongly diminished amounts of CagL overall and in the insoluble and outer fractions, had the most severely impaired cell activation phenotypes with reduced IL-8 induction values of around 10% of the wild type values in the case of SU2 CagL^ΔLDQNT^ (Fig. 3, Table 1). These latter values were even lower than in the CagL-deficient negative control. SU2 CagL^ΔRGD^ induced moderately diminished cellular IL-8 values of around 70% compared to the SU2 wild type. When we combined moderately IL-8 deficient motif deletion mutants to generate double mutants (CagL^ΔTSPSAΔRGD^, CagL^ΔRGDΔTASLI^), we determined a stronger (additive) defect in IL-8 secretion than for the respective single mutants ΔTSPSA and ΔTASLI (Fig. 4). However CagL^ΔTSPSAΔRGD^ was not significantly different from CagL^ΔRGD^. Next, for testing a second known functionality of the *cag*PAI T4SS, we examined the translocation of the CagA effector protein into host cells by coculturing *H. pylori* SU2 wild type and all *cagL* SDM mutant strains (two clones of each strain) with AGS cells. CagA translocation and intracellular phosphorylation, determined as the amount of pTyr-CagA upon cell coincubation, are completely dependent upon the presence of CagL^22^. The results are summarized in Table 1. The SU2 CagL^ΔNNEM^ mutants displayed no reduced CagA translocation (densitometry quantitation normalized to the wild type used in the respective experiments; Methods). In SU2 CagL^ΔRGD^, CagL^ΔTSPSA^ and SU2 CagL^ΔTASLI^ mutants, that had a moderate deficiency in IL-8 secretion, we noted a comparable reduction of CagA translocation to about 55 to 70% of the parental strain (Supplementary Fig. S6). SU2 CagL^ΔELFFI^ and SU2 CagL^ΔFKTYM^, which displayed an intermediate phenotype in CagL expression levels, with reduced CagL in the outer bacterial fraction, and intermediate IL-8 induction, showed a strongly reduced (<10% pTyr-CagA) CagA translocation phenotype, while the mutants deleted in the interspersed motifs SEDVI and AEQQC were considerably less deficient (ca. 30% pTyr-CagA) in comparison to the wild type (Supplementary Fig. S6). We did not detect any pTyr-CagA at all in AGS cells co-cultured with SU2 CagL^ΔLDQNT^ and SU2 CagL^ΔGISDK^ (Supplementary Fig. S6), both located in loop 3 and which had strongly reduced CagL and no detectable CagL in the outer fractions.

### *H. pylori* CagL motif mutants influence CagI amounts and CagI localization in subcellular bacterial fractions to a different extent, but have no major influence on CagA, CagH and CagY

In a recent study, three additional *cag*PAI-encoded proteins (CagA, CagI, CagY) have been identified which, in addition to CagL, can interact with host cell integrins^33^. CagL, CagI and CagY might thus jointly contribute to CagA translocation. Furthermore, several studies demonstrated that CagI and CagH are two other essential components of the Cag T4SS that, jointly and in close interaction with CagL, might form a sub-complex at the surface of the bacteria^24^,^25^. We therefore tested whether CagL amounts in our set of motif deletion mutants influenced the amounts of CagA, CagY, CagI, or CagH, which might help explain a decrease in CagT4SS function.

We did not detect a major influence in bacterial lysates of all *H. pylori* CagL motif deletion mutants on overall CagA amounts (+/− 13% differences in normalized band intensity detected by densitometry; Fig. 2), or on the distribution of CagA in the three tested subcellular bacterial fractions (Supplementary Fig. S5; and data not shown). CagY amounts and localization were also not changed in any of the mutants (data not shown).

While we detected CagI to varying amounts in the motif deletion mutants (Fig. 2; Supplementary Fig. S7; and see above), in most mutants directly proportional to the amounts of CagL, the mutations did not have a major influence on CagH amounts (maximal deviation from the wild type in densitometry of approximately 25%; Fig. 2; Supplementary Fig. S7). In the fractionation experiments, we detected CagI mainly in the outer and insoluble fractions in the *H. pylori* SU2 parental strain, with a minor portion present in the soluble fraction (Supplementary Fig. S5). The negative control, *cagL* insertion mutant (SU2 *cagL*::*km*), exhibited strongly reduced CagI amounts in both the outer and the insoluble fractions. Interestingly, CagI showed, in addition, a slight molecular mass shift in the absence of CagL (Supplementary Fig. S5). Fractionation analyses of the *cagL* motif mutants revealed differential effects of the mutants on CagI localization, depending on the site of CagL motif deletion: deletion mutants in the hydrophobic CagL region (α2 and adjacent loop region 2; Fig. 1B), SU2 CagL^ΔELFFI^ and SU2 CagL^ΔFKTYM^, that had already exhibited overall reduced CagL and CagI levels (Fig. 2), also revealed that CagI levels were decreased in all fractions (Supplementary Fig. S5). However, while remarkably in the SU2 CagL^ΔELFFI^ mutant, CagI was not detected in the bacterial outer and soluble fractions, SU2 CagL^ΔFKTYM^ still showed, albeit reduced, CagI amounts in the outer fraction. Concerning CagL motif deletions in loop region 3 (Fig. 1B), which had the most substantial loss of CagL, CagI was most strongly decreased in all analyzed fractions (most notably in the insoluble fraction) in mutant strain SU2 CagL^ΔLDQNT^, which exhibited strongly impaired amounts and subcellular distribution of CagL and a complete deficiency to activate AGS cells. In the same loop region, adjacent mutant SU2 CagL^ΔAEQQC^ also showed overall reduced CagI, despite having only slightly reduced CagL. In the last mutant of the same loop, SU2 CagL^ΔGISDK^, despite the remarkably CagL-deficient phenotype of this mutant (Fig. 2), CagI was still detected, however in moderately reduced amounts, in all fractions (Supplementary Fig. S5; Table 1). This suggests a priority of CagI and partial uncoupling of CagL and CagI in all mutants in loop 3. The chromosomal SU2 CagL^ΔRGD^ mutant did not show any remarkable change in CagI distribution compared to the parent (Supplementary Fig. S5). The three remaining CagL motif mutants did not show a strong effect on CagI amounts or localization (Fig. 3; Table 1; Supplementary Fig. S7).

### Differential binding of purified CagL variants to integrins and gastric epithelial cells

**Figure 5:**
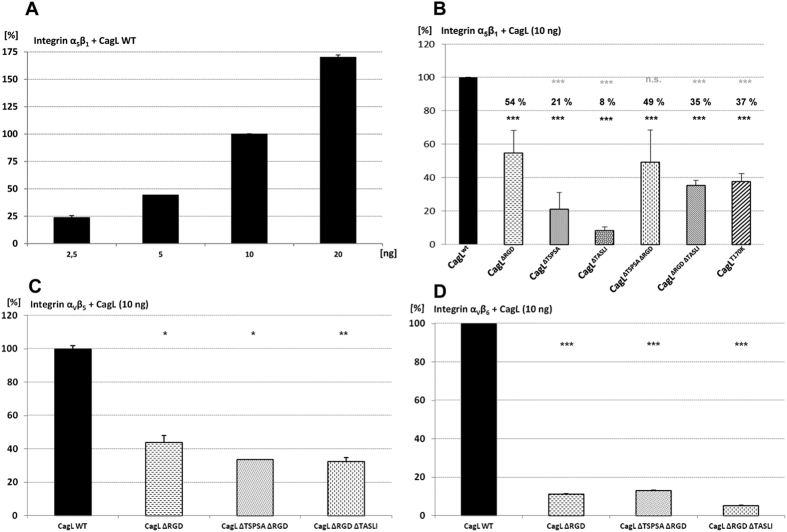
Differential binding of different site-directed CagL motif mutants to recombinant human integrins α5β1, αvβ5 and αvβ6. Highly purified native CagL motif mutant proteins (ng amounts, as indicated) were incubated in microwell plates coated with recombinant human integrin heterodimers (R&D Systems) for 3 h (followed by extensive washing) and quantitated by an ELISA-like sandwich method using anti-CagL antibody (Methods). The results are depicted as relative values in comparison to the purified CagL wild type control, which was set to 100% (Methods). In (**A**), the concentration-dependent binding of CagL wild type to integrin is shown; in (**B**,**C**,**D**), the binding of wild type CagL and different CagL mutants (10 ng each) to different integrin variants (α5β1, αvβ5, αvβ6, respectively) is depicted. A significant loss of binding of the mutated CagL variants in comparison to the wild type was quantitated for each integrin type. In panel B, mean values and significances of differences towards CagL wild type binding were calculated by student’s *t*-test (two-tailed, unpaired) from the mean and SD of three independently performed experiments, summarizing seven data points in total for each protein, and are shown as black asterisks above each bar: ***p < 0.01. Upper row of asterisks in (**B**) significance of differences between CagL^ΔRGD^ mutant and other mutants, respectively, calculated on the basis of the same experiments. In panels (C) and (D), mean and p values from technical duplicates of one experiment, respectively, are depicted. n.s. = not significant.

As shown above, several *H. pylori* motif deletion variants in CagL revealed a significantly deficient phenotype in cell activation assays, while the amounts of CagL or CagI seemed not altered. This suggested that these mutated segments fulfil a function in host cell interaction. In particular, the ΔTSPSA and ΔTASLI motif deletion mutants fell into this category, indicating a functional deficiency of the protein combined with structural integrity. In addition, the two motifs are relevant to the positioning of the RGD motif and might influence integrin binding: the first motif (TSPSA) is located in the disordered region between the α1 and α2 helices (loop 1) and may therefore impact on the positioning of the two helices with respect to each other^32^ (Supplementary Fig. S4); the second motif (TASLI) is located between helices α5 and α6 (loop 4) directly opposite the RGD motif (Fig. 1B) and may influence RGD-dependent binding directly or indirectly. This encouraged the hypothesis that both these motifs may act in concert with the RGD motif on cells. In order to reveal a functional role and potential redundancy of some of these motifs with regard to cell interaction, we established binding assays (ELISA-like assays) in a multi-well format to determine the interaction of purified CagL variants with integrins and human gastric epithelial cells (Methods). We recombinantly expressed and natively purified CagL wild type protein and selected CagL motif variants in a two-step procedure to larger than 98% homogeneity (Supplementary Fig. S8). In addition to wild type protein, we chose the CagL^ΔRGD^ motif variant as a control for these assays, since this motif was previously reported to be involved in integrin binding^27^,^29^,^32^,^41^. We also added the CagL^ΔTASLI^, CagL^ΔTSPSA^, and the CagL^ΔRGDΔTASLI^ and CagL^ΔRGDΔTSPSA^ double motif deletions to the tested panel of purified CagL proteins (Supplementary Fig. S8). We first compared wild type CagL and the other CagL motif variants for integrin binding. CagL integrin binding was concentration-dependent (Fig. 5). While the CagL^ΔRGD^ motif mutant, as expected, had a significantly reduced binding phenotype to various integrins in comparison to wild type CagL (<50%) (Fig. 5), we determined an even stronger reduction of integrin binding for the CagL^ΔTASLI^ (<10%) and CagL^ΔRGDΔTASLI^ (<36%) variants (Fig. 5). Interestingly, the single CagL^ΔTASLI^ variant already showed a strongly deficient phenotype, even less binding than the double-motif-deficient protein CagL^ΔRGDΔTASLI^. The TASLI motif therefore seemed to contribute to integrin interaction. The polar threonine residue T170 of TASLI is located directly opposite the RGD aspartate D78 in the CagL structure, suggesting a functional interaction of both residues. In order to elucidate its relevance for CagL integrin binding, we constructed a single CagL substitution mutant, T170K, replacing threonine 170 with lysine, which results in altered physicochemical properties at this site (K170 being a potentially positively charged amino acid with a predicted local pKa between 7 and 8 (Methods)). The T170K mutant, when expressed chromosomally in *H. pylori*, had a slight but significant effect (reduction by about 20%; Fig. 4), similarly to the ΔTASLI mutant, on IL-8 secretion, but did not lead to a significant reduction in CagA translocation when compared to the parental reference strain (Supplementary Fig. S6). When we tested the recombinantly purified CagL T170K variant for integrin binding, it also showed a significant binding deficiency at the tested conditions (Fig. 5). A CagL T170A single substitution mutant of threonine with non-charged alanine also displayed a decrease in integrin interaction, however less pronounced than the T170K mutant (data not shown). A CagL^ΔTSPSA^ mutant showed a strongly reduced binding phenotype to integrins, lower than the CagL^ΔRGD^ and similar to the CagL^ΔTASLI^ mutant (Fig. 5). The CagL^ΔRGDΔTSPSA^ double deletion mutant did not mediate a further reduced integrin interaction in comparison to CagL^ΔRGD^. With regard to cell activation by live bacteria, the SU2 CagL^ΔRGDΔTASLI^ double motif mutants led to a more strongly diminished IL-8 secretion upon coincubation with AGS cells than the single CagL^ΔRGD^, CagL^ΔTASLI^ and CagL^ΔTSPSA^ mutants (to about 60% of the wild type levels; Fig. 4), while the CagL^ΔRGDΔTSPSA^ mutant was inducing IL-8 secretion at the same level as CagL^ΔRGD^. This result indicated a very minor involvement of the TSPSA motif in IL-8 induction in the absence of RGD.

**Figure 6:**
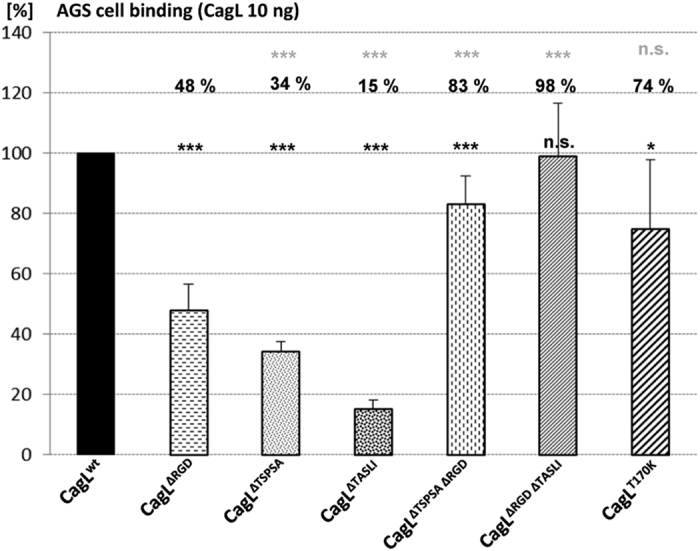
Differential binding of site-directed CagL mutants to AGS human gastric epithelial cells. Highly purified native CagL variants (ng amounts, as indicated) were incubated in microwell plates containing fixed adherent AGS cells for 3 h, followed by extensive washing, and quantitated by an ELISA-like method using anti-CagL antibody (Methods). The results are depicted as relative values in comparison to the CagL wild type control, which was set to 100%. Cell binding by different CagL mutants (10 ng each of highly pure protein) in comparison to wild type CagL is shown. Significances of differences to CagL wild type binding were calculated by student’s *t*-test (two-tailed, unpaired) from the mean values and SDs of three independently performed experiments, summarizing six data points in total, and are shown as black asterisks above each bar: ***p < 0.01; *p < 0.05. Upper row of asterisks: significance of differences between CagL^ΔRGD^ mutant and other mutants, calculated on the basis of the same experiments. n.s. = not significant.

In a similar assay, we assessed the binding of purified CagL^wt^ protein variants to fixed AGS cells. CagL^ΔRGD^ resulted in significantly reduced binding to cells, again to about 50% of CagL^wt^. CagL^ΔTSPSA^ (34% of wild type protein) and CagL^ΔTASLI^ (15% of wild type protein) mutants bound significantly less well to cells than the wild type protein and the CagL^ΔRGD^ mutant protein. However, both double deletion proteins, CagL^ΔRGDΔTSPSA^ and CagL^ΔRGDΔTASLI^, showed no further reduction in AGS cell binding in comparison to the CagL^ΔRGD^ single motif mutant (Fig. 6). In contrast, the purified CagL^ΔTSPSAΔRGD^, CagL^ΔRGDΔTASLI^ and CagL^T170K^ mutated proteins bound significantly better to cells than the CagL^ΔRGD^ single mutant. This suggested that the TASLI motif contributes to integrin binding, while the same motif, probably in a conformation-dependent manner, is not required for efficient CagL cell binding in the presence of other potential cellular binding partners (Discussion). Under conditions of reduced pH (≤ 6) during cell binding, we determined an even more increased binding of CagL^T170K^, CagL^ΔTASLI^ and CagL^ΔRGDΔTASLI^ proteins to AGS cells in comparison to the wild type protein (data not shown; see Discussion).

## Discussion

CagL, which was described as a possible VirB5 ortholog of the *H. pylori* CagT4SS^21^, is essential for the function of the CagT4SS^22^. CagL is a secreted protein of the Cag apparatus which stays at the bacterial surface, where it can interact with cellular factors, unlocking the functionality of the CagT4SS^24^,^27^. One such established cell attachment factor is human integrin. Integrin can be bound by the RGD motif in CagL^27^,^29^,^32^ and RGD-independently by other *cag*PAI proteins^33^, and is involved in CagA translocation. Based on 3D structural modelling of CagL loop regions, confirmed by several recently clarified crystal structure variants of CagL^32^,^37^,^38^,^42^, the analysis of global *H. pylori* inter-strain variation, and the comparison of *H. pylori* CagL with other potential bacterial orthologs, we have formed the hypothesis that unique loop regions in CagL harbor as yet unknown functions. We have therefore aimed in the present study at defining novel CagL motifs which can be involved in functions of CagL with regard to the CagT4SS and host cell interaction.

Taken together, we have confirmed the hypothesis that CagL-specific loop regions, in particular sequence-variable ones, can exert novel functions with respect to CagL and the host interaction of Cag T4SS. Through detailed analysis of a set of ten chromosomal CagL motif deletion mutants in *H. pylori*, we found that several motifs, localized predominantly in disordered regions, play novel roles in maintaining distinct CagL protein functionalities. We have pinpointed amino acids at the edge of helix α2 (CagL core region and loop 2) and a large loop region between the α3 and α5 helices (loop 3) that impact on CagL amounts. The same segments also codetermine CagI amounts and are possibly involved in CagL interaction with the Cag pilus protein CagI. We have also found that two novel strain-variable CagL loop motifs, TSPSA^5^,^34^,^35^,^36^ in loop 1, and TASLI (aa 170 to 175 in loop 4), are involved in determining the strength of integrin interaction (TSPSA and TASLI), or modulate CagL cell binding (TASLI). The first of these latter CagL segments has been previously highlighted: several research groups have determined an association between amino acid polymorphisms in this segment adjacent to TSPSA (in particular in amino acids 58 and 59) and the cancer risk of infected patients^34^,^36^,^43^. There is conflicting evidence on the role of these amino acids, and the pathological association of polymorphisms has not been causally confirmed yet.

In the present study, we have functionally analyzed those ten CagL motif deletion mutants for CagL integrity, bacterial localization and host cell interaction. Possibly related to CagL protein integrity, we have identified three motifs at the edge of helix α2 which strongly contribute to overall amounts of CagL in the cell but hardly impair CagL subcellular localization. We propose that these three motifs, ELFFI, SEDVI and FKTYM, mainly contribute to CagL stability and integrity (Fig. 1B; Supplementary Fig S3). This hypothesis, which was raised earlier by others^31^ is supported by the CagL crystal structure that revealed that these motifs are located within the hydrophobic CagL core, which is presumably required for proper protein folding and stability^31^,^38^. Moreover, the deletions of either the ELFFI or FKTYM motifs contributed to a directly proportionate loss of the CagL-interacting Cag pilus protein CagI^24^,^25^, which may offer additional explanations for the strong loss-of-function phenotype of these variants in host cell interaction (IL-8 secretion and CagA translocation). CagL and CagI have previously been shown to directly interact with each other and to contribute jointly to the formation of Cag-dependent pili-like structures upon host cell contact^24^,^25^. The loss of these structurally important CagL motifs may lead to misfolding of CagL in the bacterial cytoplasm or periplasm. Hence, this change may contribute to low amounts of persisting CagL protein and also bring about the loss of the partner protein CagI.

Deletion of either of two other motifs of a second novel functional category (LDQNT and GISDK in CagL loop 3) led to an even stronger reduction in CagL amounts in *H. pylori*, accompanied by an almost total loss of CagL in the soluble and outer fractions. *H. pylori* mutants carrying either of these two motif deletions had severely impaired phenotypes for IL-8 induction and, in particular, an almost complete loss of CagA translocation. The CagL^ΔGISDK^ mutant, while maintaining some CagI in all fractions, albeit at low levels, had a transport defect of CagL to the outer fraction, while the CagL^ΔLDQNT^ mutant had a deficiency of CagI in the insoluble fraction. We therefore speculate that those loop 3 motifs may involve a direct interaction of this CagL protein domain with CagI^24^,^25^, possibly during membrane passage, and assist in the secretion pathway of CagL to the bacterial surface. It is not excluded that, in addition, the lack of LDQNT and GISDK motifs leads to misfolding and some degradation of both CagL and CagI. Deletion of the loop 3 interspersed motif AEQQC reduced CagI but not CagL amounts, offering a further argument that this segment might be involved in CagL-CagI cooperation.

In general, a reduction in CagL amounts led to a proportionate reduction of CagT4SS function, even if the loss was relatively minor and CagL still localized correctly, for instance in the mutants CagL^ΔELFFI^, CagL^ΔSEDVI^ and CagL^ΔFKTYM^. With two exceptions, CagL^ΔSEDVI^ and CagL^ΔAEQQC^ bacteria, both CagI amounts and T4SS activities in the mutants appeared to be directly proportionate to CagL amounts.

Furthermore, we have identified a set of other motifs of a third novel functional category in CagL which did not lead to a major loss of CagL expression nor impaired subcellular localization of CagL and its interacting proteins, and exhibited a moderate but significant functional reduction of the CagT4SS in the context of live bacteria and AGS cell coculture. We considered these motifs particularly interesting, since they might be involved in modulating CagL and *cag*PAI activities with respect to cell interaction. Two of these motifs, the well-studied RGD motif (a known integrin binder^27^,^28^,^44^) and TASLI, are located closely adjacent and opposite each other in the CagL structure^37^. Mutations in the CagL RGD motif revealed before that it is involved in inducing cell signalling and NF-κB activation via integrins, even in the absence of CagA translocation^44^. Purified CagL deletion variants ΔRGD and ΔTASLI independently had a clearly deficient phenotype in integrin binding. Although expected for the RGD motif deletion, the ΔTASLI deletion surprisingly showed an even more drastic loss of native integrin binding with all tested integrins. The RGD- and TASLI-dependent loss of CagL binding to integrins was particularly severe for integrin α_V_β_6_, which has recently been shown to possess a particularly high affinity to CagL^28^. We therefore propose that the TASLI motif is a novel trait that contributes to integrin binding independently of RGD. In addition to an independent function, it may also contribute, presumably by stabilizing the conformation of the opposing RGD and TASLI regions within the CagL tertiary structure, to better positioning the RGD motif for host cell integrin binding. Additional assays (own unpublished data) revealed that the phenotype of integrin and cell binding of CagL was also influenced by the composition of the blocking buffer, in particular by the presence of serum proteins. Since the integrin-binding protein collagen^45^ was able to partially mimick this serum effect (own unpublished data), which was lost for the ΔTASLI mutant, we currently propose that the TASLI motif contributes indirectly to the binding of integrins in the presence of integrin-binding serum protein. Another motif deletion reducing the binding of purified CagL to integrins was TSPSA in loop 1. Our results suggest that this effect is not direct, but possibly introduces a conformational change that may alter the positioning of CagL alpha helix α2. Template-based structural modelling of the CagL TSPSA deletion mutant also suggested a slight repositioning of the α2 helix with regard to α1 and the intrinsic turn of helix α2. In line with this, recent structural data^32^,^42^ support the hypothesis that the position of α2 and the relative orientation of helix α2 versus helix α1 are important for the RGD motif to be in the correct orientation to optimally interact with integrins. This conformational flexibility impacts on CagL integrin binding, in particular at low pH as recently demonstrated^32^. Another RGD helper sequence (RHS), the amino acid motif “FEANE”, was reported which influences the binding of the neighboring RGD motif to integrins^30^. We did not include this RHS in our analyses, since it is located in the stabilizing hydrophobic core region (α2) of CagL^37^ and is not subject to diversifying selection in the CagL sequence^5^. The *H. pylori* strain variability in and downstream of the TSPSA region, with short deletions of several amino acids prevalent in different geographical isolates^5^ (Supplementary Fig. S1) also invites the hypothesis that, with regard to host interactions, evolutionary selection might have modulated CagL structure towards an optimized function of CagL in the respective host ethnicities. This hypothesis cannot easily be verified *in vitro*. Transferring certain mutations in this region to a reference strain and testing these on host cells as has in part been done already^46^ might offer more clues as to whether these variations modulate the interaction of CagL with host cells. In addition, strain-specific structural differences in CagL variants, for example disulphide bridges, which can confer higher stability at lower pH^42^ are suggestive to be the result of functionally diversifying evolution.

While we determined a strongly reduced phenotype in CagL integrin binding of the CagL^ΔTASLI^ and ΔRGDΔTASLI double mutants, the effect of mutated live bacteria on CagA translocation and IL-8 secretion, even if the two motifs were deleted in combination, was relatively minor (max. 40% inhibition for the ΔRGDΔTASLI double deletion mutant). Notably, while a significant reduction in binding of purified CagL to fixed AGS cells was shown for the ΔRGD and even more so for the ΔTASLI deletion, the combined deletion of RGD and TASLI had a close to wild type phenotype for CagL binding to gastric epithelial cells. This effect was even enhanced at lower pH (own unpublished data). These findings also corroborated earlier reports^33^,^30^ that the RGD motif is not essential for CagA translocation and IL-8 induction. This indicates that these effects can occur by integrin-independent interaction of CagL with cells. It may also be fair to speculate, that in contrast, if integrin binding might be absent by cell type characteristics, or if a pH shift, involving CagL conformational change, might diminish integrin binding^32^, other cellular factors might come into play for CagL binding and T4SS functionality. This also supports the idea that, in addition to dedicated integrin binding segments, other CagL sections are likely involved in interaction with the cell surface and cell activation for T4SS effector translocation.

Indeed, alternative CagL crystal structures obtained at a pH below 5 demonstrated that, in addition to helices α1 and α2, helices α5 and α6 may undergo a conformational change^28^,^32^. The disordered region between helix α5 and α6 apparently can convert to an extended helix in the place of the antiparallel α5 and α6 fold^28^,^32^. The TASLI motif is localized in the extended conformation in the center of the extended helix with a different tilt, and also the orientation of the TASLI motif towards the RGD motif in the adjacent helix α2 will be changed. While Barden *et al*. showed that T170 and D78 of the two structurally opposing motifs (RGD, TASLI) are positioned opposite each other with a short distance at close to neutral pH^37^, the crystal structures obtained at lower pH^28^,^32^ reveal a conformational separation of the two helices and hence of the two motifs. Our results appear to indicate that upon the joint deletion of TASLI and RGD, CagL may undergo a conformational change. This still needs to be confirmed by biophysical methods in the future. Assuming that these deletions bolster an elongated conformation of helices α5 and α6, they may contribute to a stronger CagL cell binding, while possibly diminishing integrin binding. A similar phenotype as for the ΔRGDΔTASLI mutant was also observed when T170 in TASLI was mutated to lysine. This predicted potentially pH-sensitive mutation (Methods), which might affect the electrostatic interaction with the opposite D78 residue, might increase the propensity of the α5 and α6 helices to flip to an extended conformation. Altogether, these results encourage the hypothesis that a novel integrin-independent cell-binding patch within CagL is exposed upon the structural elongation of the α5 and α6 helices.

Only two proposed VirB5 proteins of T4SS have been structurally characterized so far, TraC, which is a remote ortholog from a DNA conjugation system in *Escherichia coli*^26^ and CagL^37^,^42^,^32^. While TraC^26^ and CagL^37^ are not very similar in structure, they exhibit common features such as a high content in alpha helices, arranged in an antiparallel conformation. In both these proteins, these alpha helices are interspersed with disordered regions, whose functional importance is underlined by our current characterization of the CagL loop segments. The best characterized host-directed T4SS translocation systems include the ones from *A. tumefaciens* (plant-directed^21^,^47^), *Bartonella* spp. (mammalian hosts^48^), and *Brucella* spp. (mammalian hosts^49^,^50^). When analyzing those proposed VirB5 proteins, which we modeled using CagL as a template, the *A. tumefaciens*, *Bartonella* and *Brucella* proteins possess predicted loop regions between their antiparallel alpha helices (e.g. corresponding to loops 1 and 4 in CagL, sequence alignment shown in Supplementary Fig. S2). With regard to our present findings for CagL, those specific loop regions (Supplementary Fig. S9) may be instructive for investigating interactions with their respective target bacterial or host cells.

Taken together, we have characterized three novel categories of functionally important CagL motifs. Two types of deletions led to strongly diminished Cag T4SS function, by reducing the amounts of both CagL and CagI: one category of motifs located at the edge of helix α2 (loop 2) likely is important for CagL integrity and stability, while the second segment (in loop 3) may be involved in direct CagL-CagI interactions. Most importantly, we have identified two other chromosomal motifs in addition to RGD (TSPSA in loop 1 and TASLI in loop 4) whose deletions formed stable variants of CagL and do not abolish CagL expression or proper localization. These two motif mutants have only a moderate defect in IL-8 secretion and CagA translocation in human gastric epithelial cells after *H. pylori* coincubation, however the motifs appear to strongly contribute to integrin and cell interactions. For both, TSPSA, and the TASLI motif, structurally opposite RGD, we were able to show for the first time that they are involved in CagL integrin interaction. The TSPSA loop is the most strain-variable region in CagL, including amino acid deletions, in particular in East Asian strains. We propose that TSPSA is surface-associated in CagL and contributes to the positioning of the RGD motif which indirectly affects integrin binding. TASLI contributed strongly to CagL integrin binding even independently from RGD, while the absence of TASLI and RGD together even enhanced CagL cell binding. Interestingly, only very few other *H. pylori* proteins contain highly conserved TASLI motifs (own unpublished data). Most notably, ImaA (HP0289), an autotransporter protein and VacA ortholog of *H. pylori*^51^, contains one complete and a second, incomplete (ASLI) motif. ImaA was previously reported, possibly via competition for host integrin, to compete with the Cag T4SS for cell interaction and to reduce *cag*PAI-mediated cell activation *in vitro* and *in vivo*^51^. In conclusion, variable loop regions of CagL exhibit *H. pylori*-specific, novel T4SS functions. Some are involved in RGD-accessory integrin binding, or in possibly integrin-independent host cell interaction. These findings confer the potential to inhibit Cag T4SS function and may encourage novel approaches to tackle the structural and functional characterization of potential orthologs in pathogenic bacteria.

## Materials and Methods

### Bacterial strains and culture conditions

*H. pylori* parental strains 26695^52^, and SU2^5^ were used for genetic manipulations, protein analysis and eukaryotic cell infections. The two strains harbour a functional *cag* pathogenicity island (*cag*PAI) in their genome, encoding the Cag T4SS^4^,^5^. *H. pylori* strains and mutants used in this study are listed in Supplementary Table S1. Bacteria were regularly grown on blood agar plates (Oxoid blood agar base II, Wesel, Germany) supplemented with 10% (v/v) horse blood and the following antibiotics: amphotericin B (4 mg/liter), polymyxin B (2,500 U/liter), trimethoprim (5 mg/liter) and vancomycin (10 mg/liter). Plates were continuously incubated in anaerobic jars under microaerobic atmosphere generated by Anaerocult C sachets (Merck, Darmstadt, Germany). Mutant strains were selected and cultivated on blood agar plates mixed with chloramphenicol (10 mg/liter) and/or kanamycin (100 mg/liter). *E. coli* strains DH5α, MC1061, XL1-Blue and XL10-Gold were used for cloning experiments (Supplementary Table S1). For the recombinant expression of *H. pylori* CagL, *E. coli* BL21(DE3) was used. *E. coli* strains were propagated on Luria Bertani plates or in LB broth containing ampicillin (200 mg/liter), chloramphenicol (20 mg/liter) and/or kanamycin (100 mg/liter) as required.

### Standard techniques for DNA and protein analysis

Standard procedures for cloning and DNA analysis were performed according to^53^. DNA modification and restriction enzymes were purchased from Invitrogen (Life Technologies), New England Biolabs (NEB) or Roche. Isolation of highly pure genomic DNA, purification of plasmid DNA and gel extraction of DNA from enzymatic reactions were accomplished by QIAGEN DNA purification columns (QIAGEN, Hilden, Germany). Plasmids and oligonucleotide primers for cloning, sequencing and site-directed mutagenesis are depicted in Supplementary Tables S2, S3 and S4, respectively. PCRs were run in Biometra thermocyclers (Biometra, Goettingen, Germany) using *Taq* polymerase (GE Healthcare). *Pfu* (Agilent Technologies) and Phusion (NEB) proofreading polymerases were used for accurate amplification of longer DNA fragments. Protein separation and detection by denaturing SDS polyacrylamide gel electrophoresis (SDS PAGE) and Western blotting were performed according to standard methods^54^,^55^. Total protein concentration was measured using the bicinchoninic acid (BCA) assay (Thermo Scientific). Protein concentration and purity of recombinant proteins were estimated by SDS PAGE and Coomassie or silver staining, comparing the yield to defined BSA standards. Protein samples were separated on 10.6% or 11.5% SDS polyacrylamide gels depending on the molecular mass of the protein to be analyzed. Subsequently, protein samples were transferred to nitrocellulose membranes (Schleicher & Schuell, Germany) by tank blotting. Antisera and antibodies for immunodetection were diluted in TBS-T (TBS with 5% skim milk and 0.1% Tween 20) as noted in the figure legends. Secondary antibodies were used as conjugates to horseradish peroxidase (HRP). For detection of the HRP signal, membranes were incubated with enhanced chemiluminescent substrate (SuperSignal West Pico, Thermo Scientific) and exposed to chemiluminescence films (Amersham Hyperfilm^TM^ ECL, GE Healthcare). Before reapplication of antibodies, membranes were stripped with Restore^TM^ Western Blot Stripping Buffer (Thermo Scientific).

### Cell fractionation and isolation of *H. pylori* surface-exposed proteins

Whole cell lysates were generated from bacteria grown for 1.5–2 d on blood agar plates and resuspended in Tris/HCl (100 mM, pH 7.5). Bacteria were sonicated three times for 2 min and the protein content of the lysates was determined. Equal amounts of protein for each preparation were separated on SDS gels for comparison. For the quantification of CagL, CagI, CagH, and CagA protein amounts, the detected signal intensities from Western blots were scanned to TIFF files and analyzed by densitometry using ImageJ^56^. Protein amounts (band intensities) were normalized to invariable bacterial protein bands detected by an anti-*H. pylori* polyclonal antiserum (DAKO; 1:10,000).

For the subcellular localization of wild type or mutated CagL, bacteria were separated into outer (surface-enriched), soluble and insoluble fractions by bacterial shearing, sonification and differential centrifugation. Preparation of the bacterial outer fraction (OF), containing surface-exposed proteins and bacterial appendages, was accomplished as previously described^19^. In brief, cells were grown for 1.5–2 d on blood agar plates, resuspended in Tris/HCl (100 mM, pH 7.5) and sheared by pushing the suspension repeatedly (30 times) through 26-gauge syringe needles (Braun Sterican^®^, 0,45 × 25 mm, Braun, Melsungen, Germany) to detach surface-exposed material. Intact bacterial cells were separated from the sheared material by repeated differential centrifugation steps (21,912× g for 20 min, 186,000× g for 1 h in an Optima CU-TL100 Beckman ultracentrifuge). Remaining bacterial pellets were resuspended again in Tris/HCl (100 mM, pH 7.5), supplemented with Complete Mini protease inhibitor cocktail (Roche) and sonicated for 1–2 min at 4 °C (Branson Sonifier 450, setting 5). Subsequently, the lysates were separated into soluble (enriched in cytoplasmic and periplasmic material) and insoluble fractions (membrane-enriched) by centrifugation (186,000× g, 30 min, 4 °C). The supernatants (soluble fractions) were removed and the pellets, containing the crude membrane (insoluble) fractions, resuspended in Tris/HCl (100 mM, pH 7.5, Complete Mini). All prepared fractions were analyzed by Western immunoblotting. CagL running at approximately 26 kDa was detected using α-HP0539 antiserum AK271 (Fig. 2)^22^. CagI was detected at approximately 40 kDa using α-HP0540 antiserum (Fig. 2) that was described previously^25^. Cell fractionations were verified by an antiserum raised against *H. pylori* FlhA (membrane-bound component of the flagellar protein export apparatus) as a fractionation control for the insoluble fraction (Supplementary Fig. S5). As a fractionation control for the outer fraction, an antiserum against the *H. pylori* outer membrane protein HopZ was used, which detected HopZ exclusively in the insoluble (membrane-enriched) fractions (data not shown) (antiserum polyclonal rabbit α-HopZ/type1, 1:10,000)^57^,^58^.

### CagL *in silico* analyses

For the CagL protein secondary and tertiary structure predictions, we used the CagL primary sequence from strain 26695^52^ as query sequence. The CagL signal peptide was predicted by SignalP 4.1^59^, the secondary structure by JPred3 (incorporating Jnet)^60^. For the structure-based 3D structural modelling of CagL, we used the PHYRE protein homology/analogy recognition engine and database^61^,^62^. CagL 2D and 3D structural predictions were performed before the first CagL structure was published to define the proposed CagL leader peptide segment for cloning purposes, and to instruct the definition of disordered and potentially instable regions. In silico predictions were also used throughout the work to assess the potential structural stability of protein regions or mutated proteins. For the template-based structural prediction of CagL mutant proteins, we used the online structure homology modelling server Swiss Model^63^.

In order to support the design of single substitution mutants in CagL, we used in silico prediction of local pKa of charged amino acid residues. We thereby determined the propensity of K170 in the CagL substitution mutant T170K to be locally charged under different conditions, which might be relevant for potential conformational change. The local pKa values for all lysine residues of CagL variant T170K were analyzed based on the CagL protein structure with DEPTH Server^64^. The results suggest that all other lysine residues except for “artificial” lysine 170 in CagL possess pKa values of around 10 (as expected). For substituted lysine 170, a local pKa of 8.3 is proposed by DEPTH. As a second and independent prediction software, H++ (http://biophysics.cs.vt.edu/index.php), also based on the CagL structural information, gave a similar output, suggesting a local pKa of 7.3 for K170, while all other, natural, lysine residues in CagL have a pKa of around 10. Both applications require a pdb file as input and thus take the local environment and solvent accessibility into account. Standard settings were used for these predictions, and the CagL T170K model pdb (derived from pdb:3czj_chainA) was supplied. The results appear therefore to be reliable and suggest that at physiological pH values (between 7 and 8) in the context of its protein environment, the substituted lysine 170 residue may be partially protonated and partially non-protonated.

### Recombinant CagL expression, native purification and protein characteristics

To construct a plasmid for the heterologous expression of CagL (wild type) in *E. coli*, oligonucleotide primers HP0539_fw2 and HP0539_rv2 were used to amplify the *cagL* coding region from *H. pylori* 26695 genomic DNA without the predicted CagL N-terminal signal sequence (amino acid residues 21–237). The amplified *cagL* DNA segment was ligated in-frame at *Eco*RI and *Not*I sites into the pGEX-4T-2 expression vector (GE Healthcare) yielding plasmid pCJ905. This plasmid expresses a fusion protein with an N-terminal GST tag. The nucleotide sequence of the *cagL* coding region in pCJ905 was verified by PCR and Sanger sequencing. For the heterologous expression of mutated CagL motif variants, plasmids were constructed analogously with the mutated sequence.

For expression cultures, 200 ml Luria broth were inoculated with an overnight culture of *E. coli* BL21(DE3) transformed with the respective plasmid (Supplementary Table S2). Bacteria were grown in a shaking incubator at 150 rpm, 30 °C. CagL expression was initiated at an OD_600_ of 0.6 by the addition of 0.2 mM IPTG. Growth of the cultures was continued for four additional hours. Bacteria were harvested by centrifugation (6,000× g, 10 min).

Purification of GST-tagged proteins was performed as follows: In brief, bacterial cell pellets were resuspended in ice-cold PBS (Dulbecco) supplemented with protease inhibitor cocktail (Roche). Crude lysates were separated by sonication and amounts analysed by SDS gels and Coomassie staining. GST fusion proteins, predominantly located in the insoluble fraction, were solubilized in buffer S (PBS Dulbecco, 2 mM DTT, 1 mM EDTA, 0.5% sarkosyl, 0.7% Triton X-100, protease inhibitor cocktail, Roche) and clarified by centrifugation to remove insoluble cellular debris. The solubilized protein fraction was loaded onto equilibrated glutathione agarose columns (Protino^®^ GST/4B, Macherey Nagel, Germany). Subsequently to the binding, the column was washed with buffer W (PBS Dulbecco, 2 mM DTT, 1 mM EDTA, 0.1% Triton X-100, protease inhibitor cocktail, Roche). Bound GST fusion proteins were eluted in buffer E (100 mM Tris-HCl, pH = 8, 150 mM NaCl, 1 mM DTT, 10 mM reduced L-glutathione). For further downstream applications, the GST tag was cleaved off using thrombin protease (GE Healthcare) cleavage. The cleaved GST tag was removed by passing the pure proteins for a second time through a glutathione agarose column in buffer E without glutathione and collecting the pure, cleaved CagL proteins in the flow-through, while the GST stayed bound to the column. The purity of CagL after GST cleavage was then assessed on a Western blot, which was subsequently detected using anti-CagL and anti-GST antisera (Supplementary Figure S8 panel C). Prior to use, pure proteins were concentrated in 100 mM Tris-HCl, pH = 8, 150 mM NaCl, 1 mM DTT using centrifugal filter units (Amicon Ultra-15, Merck Millipore). The amounts of CagL for binding assays were carefully adjusted in ng quantities on repeated SDS gels with silver staining and respective BSA controls (See Supplementary Fig. S8). Uniform detection of each motif-deleted CagL variant with the polyclonal antiserum AK271 was verified using Western blotting of equal CagL amounts (Supplementary Fig. S8, panel C), and using also an ELISA like-setup, testing different CagL amounts of each variant in comparison.

### Construction of isogenic chromosomal unmarked mutants in *H. pylori cagL*

Plasmid constructs for the generation of isogenic chromosomal unmarked *H. pylori* SU2 *cagL* mutants (by allelic exchange) were propagated in *E. coli*. Plasmid manipulations by means of site-directed mutagenesis (SDM) were performed by inverse PCR using mutagenic primers or the QuikChange^®^ Site-Directed Mutagenesis Kit (Stratagene), using plasmid pCJ450 (Supplementary Table S2) as a template for mutagenesis. Plasmids obtained after CagL SDM are also summarized in Supplementary Table S2. Primers used for the SDM are listed in Supplementary Table S3; primers designed for the verification of mutant alleles by Sanger sequencing are contained in Supplementary Table S4. We introduced these unmarked mutations into the chromosomal copy of the *H. pylori cagL* gene (HP0539 from strain 26695) by using a sucrose-based counterselection system^65^. In brief, an *aphA3*′-III-*sacB* cassette was inserted into the *cagL* gene of strain *H. pylori* SU2 by homologous recombination (plasmid pCJ 474). Subsequent transformation with different mutated *cagL* alleles (plasmids listed in Supplementary Table S2) and counterselective growth on sucrose-supplemented blood plates enabled the selection of strains that had exchanged the *aphA3*′-III-*sacB* cassette for the unmarked mutated allele. All SDM plasmids and the *cagL* alleles of the resulting mutated *H. pylori* clones were sequenced using Sanger sequencing to verify that the correct mutations and deletions had been obtained.

### *cagL* allelic exchange mutant in *H. pylori*

For the functional characterization of CagL and potential bacterial interacting proteins, an insertion mutant in the *cagL* gene (HP0539 from strain 26695) was constructed by partial gene deletion and insertion of an antibiotic resistance cassette, followed by allelic exchange in *H. pylori*. Plasmid constructs were generated in *E. coli*. For the construction of a HP0539/*cagL* knockout mutant and further site-directed mutagenesis of selected regions, HP0539/*cagL*, HP0540/*cagI* and the C-terminal part of HP0541/*cagH* were amplified from strain 26695 using primers HP0541_F1 and HP0538_R1 and cloned into pUC18. The resulting plasmid (pCJ450) was inversely amplified using primers HP0539_F1 and HP0539_R1 and ligated at *Bam*HI sites with a kanamycin resistance cassette (*aphA3*′-III from *Campylobacter coli* from plasmid pILL600^66^, yielding pCJ451. In order to avoid polar effects, the resistance cassette was inserted in the same orientation as the target gene. Orientation of the *aphA3*′-III cassette was verified by PCR using different combinations of oligonucleotide primers (HP0541_F1 and Km1, HP0541_F1 and Km2, HP0538_R1 and Km1, HP0538_R1 and Km2) and targeted Sanger sequencing (sequencing primers available on request). Plasmids for mutagenesis and primers for cloning are listed in Supplementary Tables S2 and S4.

### Cell coculture experiments with *H. pylori* and quantification of chemokine secretion

For functional studies, *H. pylori* wt strains and *cagL* mutants were cocultured with the human gastric adenocarcinoma cell line AGS (ATCC^®^ CRL-1739^TM^). Cells were grown in RPMI 1640 supplemented with 10% fetal bovine serum (FBS) buffered with 25 mM HEPES (Biochrom, Berlin, Germany). Cell infection experiments were performed on subconfluent cell layers (60% to 90% confluence) in 24 well plates. Cells were preincubated in fresh medium with FBS 60 min prior to infection. Exponentially growing bacteria were harvested from blood plates and resuspended in RPMI 1640 (RPMI 1640, 10% FBS, 25 mM HEPES). AGS cells were infected with *H. pylori* at a multiplicity of infection (MOI) of 50. The infection was synchronized by centrifugation of the incubation plates (154× g, 10 min, 20 °C). The co-incubation time was fixed to 22 h. Non-infected cells (mock) were used as negative control. Supernatants were harvested, cleared by centrifugation (21,912× g, 10 min, 4 °C) and stored at −20 °C until further use. IL-8 secretion by AGS cells into the cell supernatant in response to *H. pylori* contact was monitored by using an anti-human IL-8 sandwich ELISA (BD OptEIA IL-8 enzyme-linked immunosorbent assay kit, BD Pharmingen, San Diego, USA) according to the manufacturer’s instructions, using appropriate dilutions. The IL-8 assays were performed in triplicate for each sample, and the cell coincubations were performed at least four times independently on different days, each time either in duplicate or triplicate infection wells. Means and standard deviations of at least four independent co-incubations measured in triplicates were calculated. The level of IL-8 secretion induced by contact with *H. pylori* SU2 wt was set to 100% and was compared to levels secreted by AGS cells after coincubation with *cagL* insertion mutants.

### CagA translocation assay

Translocation of the CagA effector protein into cells was examined by coculturing *H. pylori* strains with AGS cells. To analyse CagA translocation, AGS cells were grown in 6-well plates and infected with *H. pylori* at a multiplicity of infection (MOI) of 100 for 4 h. Non-adherent bacteria were removed by washing twice with PBS Dulbecco (pH = 7.4, Biochrom, Berlin, Germany). Cells were detached using a cell scraper and resuspended in 500 μl PBS. After centrifugation (250× g, 10 min, 4 °C) cells were lysed in 300 μl of modified RIPA lysis buffer (20 mM Tris-HCl [pH = 7.5], 150 mM NaCl, 1 mM EDTA, 1 mM EGTA, 1% Triton X-100, 2.5 mM sodium pyrophosphate, 1 mM β-glycerol phosphate, 1 mM sodium orthovanadate, 1 mM PMSF) supplemented with Complete Mini protease inhibitor cocktail (Roche) and PhosSTOP phosphatase inhibitor (Roche). During lysis, cells were placed on ice for 30 min. Cell debris was cleared by centrifugation (21,912× g, 10 min, 4 °C). The supernatant was transferred into a fresh tube. The pellet was resuspended in 100 μl RIPA lysis buffer. Both fractions were stored at −80 °C until use.

CagA translocation was evaluated by separating the soluble fractions on 10.6% SDS-PAGE (20 μg loaded per lane) and immunoblotting with an affinity-purified anti-phospho-peptide antibody (rabbit anti-*Hp*-pCagA, 1:10,000), raised against a CagA C-terminal peptide sequence CSPEPI-pY-ATID (Biosyntan, Berlin) detecting phosphorylated CagA (pCagA). Subsequently, total CagA protein (phosphorylated and non-phosphorylated CagA) was detected using a polyclonal α-CagA antiserum (rabbit α-*Hp*-CagA-antigen, IgG-fraction, Austral Biologicals, San Ramon, USA, 1:10,000). As a bacterial loading control, heat-stable *H. pylori* antigens (rabbit α-*H. pylori*, DAKO, Denmark, 1:2,500) were detected in the analyzed fractions; as a cellular loading control, we detected actin (mouse α-actin, Millipore, Schwalbach, Germany, 1:20,000). For the quantification of the pCagA protein amount, the detected signals were scanned to TIFF files (600 dpi resolution) and analysed by densitometry using ImageJ^56^. pTyr CagA (translocated CagA) was normalized for each sample as follows: First, the measured intensities of CagA signals in each lane were normalised by multiplication with a correction factor that was determined separately for each sample using the *H. pylori* invariable protein loading control (detected by rabbit α-*H. pylori*, DAKO, Denmark, 1:2,500). Secondly, the pTyr-CagA values in each lane were corrected using the normalized CagA values as percent of the respective normalized CagA positive control. Background values from the same blot comprising the same pixel numbers were subtracted from each positive band. CagA translocation assays were at least performed twice independently on different days for two clones of each mutant.

### CagL integrin binding test in multiwell plates

For the determination of the binding of CagL to one of its putative host cell receptors, integrin α_5_β_1_, purified proteins were analysed in a modified ELISA-like setup. CagL amounts for the different proteins were carefully adjusted and equalized by running multiple Coomassie-stained and silver-stained SDS gels (Supplementary Fig. S8), along with lanes loaded with an upltrapure standard protein (bovine serum albumin [BSA, Sigma-Aldrich] or glutathione-S-transferase [GST, Sigma-Aldrich]) in known concentrations. Briefly, wells were coated overnight with 100 ng/well of recombinant human integrin α_5_β_1_ or related integrins (α_V_β_5_, α_V_β_6_) (R&D Systems, Wiesbaden, Germany) diluted in coating buffer (100 mM sodium carbonate, pH = 9.5). Wells were blocked with either FCS-containing blocking buffer (FB) (10% FCS in PBS Dulbecco) or FCS-free blocker (FFB) (PBS Dulbecco, 0.2% Tween^®^20, 0.1% glycine). Subsequently, increasing amounts (2.5, 5, 10 and 20 ng/well) of recombinant CagL protein were added to also determine possible concentration-dependent effects on binding. As mock controls, wells were co-incubated with FB/FFB without CagL protein and with antiserum. As a further control, empty wells after blocking were incubated with CagL variants and antiserum to detect background attachment of CagL to the wells. This control was always lower than the mock control. The co-incubation time was fixed to 3 h. Wells were aspirated and washed repeatedly. For the detection of bound CagL, wells were incubated with α-CagL antiserum AK271 (1:10,000) and the respective HRP-conjugated secondary antibodies (1:25,000). For the detection of integrin α_5_β_1_ (control wells) wells were incubated with α-integrin β_1_-specific IgG (R&D Systems, Wiesbaden, Germany) and corresponding secondary antibodies. In some assays, collagen IV (Sigma-Aldrich) was added in the first three incubation steps, in combination with FFB. After substrate development (BD OptEIA^TM^ TMB substrate reagent set, BD Pharmingen, San Diego, USA), absorbance was read at 450 nm and 540 nm (reference wavelength) in a Titertek Multiskan^®^ MCC/340 fluorometer. CagL integrin binding assays were performed in technical duplicates or triplicates, at least three times for each mutant protein on different days. The normalization was performed after background subtraction by setting the values of the respective CagL wild type protein control to 100% and calculating relative values in percent for the respective mutant proteins (ratio of wild type CagL signal to negative background was between 8 to 10 for all experiments).

### CagL cell binding assays

To monitor the binding of purified CagL protein to AGS cells, CagL proteins were analyzed in an ELISA-like setup. CagL amounts for the different proteins were carefully adjusted and equalized by running multiple Coomassie-stained and silver-stained SDS gels, along with lanes loaded with an internal protein standard (GST or BSA) in known concentrations. Briefly, AGS cells were grown to confluence in 96 well plates (6 × 10^4^ cells/well) and fixed with paraformaldehyde (2% PFA in 100 mM potassium phosphate buffer, pH = 7). Fixing would prevent uptake and degradation of CagL by the cells. Cell fixation was stopped by the addition of quenching buffer (0.1% glycine in PBS). Fixed cells were washed repeatedly with PBS and blocked with FCS blocking buffer (FB) (10% FCS in PBS Dulbecco) or FCS-free blocker (FFB) (PBS Dulbecco, Roti-Block x0.2 [Carl Roth] 0.2% Tween^®^20, 0.1% glycine). Subsequently, recombinant CagL protein was added in increasing amounts (2.5, 5, 10 and 20 ng/well) to determine possible concentration-dependent effects on binding.

As mock controls, wells were co-incubated with FB/FFB without CagL protein. The co-incubation time was fixed to 3 h. To remove unbound protein, cells were washed with ELISA washing buffer (PBS + 0.05% Tween 20). For the detection of bound CagL, wells were incubated with α-HP0539 antiserum AK271 (1:10,000) overnight followed by incubation with the respective HRP-conjugated secondary antibodies (1:25,000). Substrate (BD OptEIA^TM^ TMB substrate reagent set, BD Pharmingen, San Diego, USA) and stop solution (1 M phosphoric acid) were added to the wells. Samples were transferred to a new 96 well plate and absorbance was read at 450 nm and 540 nm (reference wavelength) in a Titertek Multiskan^®^ MCC/340 fluorometer. CagL cell binding assays were performed in technical duplicates, at least three times for each mutant protein on different days. Normalization and calculation of relative values were done as for the integrin binding.

## Additional Information

**How to cite this article:** Bönig, T. *et al*. Systematic site-directed mutagenesis of the *Helicobacter pylori* CagL protein of the Cag type IV secretion system identifies novel functional domains. *Sci. Rep.* **6**, 38101; doi: 10.1038/srep38101 (2016).

**Publisher's note:** Springer Nature remains neutral with regard to jurisdictional claims in published maps and institutional affiliations.

## Supplementary Material

Supplementary Information

## Figures and Tables

**Table 1:** Comparative analyses of various chromosomal HP0539/*cagL* deletion mutants.

*H. pylori* strains	CagL amount	CagL secretion^a^	CagI amount	CagH amount	CagA amount	IL-8 induction in AGS cells	CagA translocation
SU2 wt	++	++	++	++	++	++	++
*cagL*::*aphA3*	−	−	+	++	++	−	**n.d.**
CagL^ΔTSPSA^	++	++	++	++	++	++	++
CagL^ΔNNEM^	++	++	++	++	++	++	++
CagL^ΔRGD^	++	++	++	++	++	++	++
CagL^ΔELFFI^	+	+	+	++	++	+	−
CagL^ΔSEDVI^	+	+	+	++	++	++	+
CagL^ΔFKTYM^	+	+	+	++	++	+	−
CagL^ΔLDQNT^	−	−	−	++	++	−	**n.d.**
CagL^ΔAEQQC^	+	++	++	++	++	++	+
CagL^ΔGISDK^	−	−	+	++	++	+	**n.d.**
CagL^ΔTASLI^	+(+)	++	++	++	++	++	++
CagL^ΔRGDΔTASLI^	−	+	+	++	++	++	+
CagL^ΔTSPSAΔRGD^	−	+	+	++	++	++	++
CagL^T^^1^^70K^	++	+	+	++	++	++	++

The plus and minus signs in each column indicate: [++], [+(+)] normal to slight reduction, [+] clear reduction, and [−] strong reduction of the respective protein or function (column header) in comparison with the parental strain (reference, SU2 wild type [wt]).

^a^The short term “CagL secretion” indicates that CagL was detected in the outer fraction of the bacteria which includes surface-associated material; CagL has been detected before by others on the bacterial surface^27^,^24^. n.d. = below detection level.
