# Supplementary material for: Systematic site-directed mutagenesis of the Helicobacter pylori CagL protein of the Cag type IV secretion system identifies novel functional domains
Source: Sci Rep. 2016 Dec 6;6:38101. doi: 10.1038/srep38101 (PMC5138618; doi:10.1038/srep38101)
Supplement: Supplementary Information [file srep38101-s1.pdf]

Supplementary Information

Systematic site-directed mutagenesis of the *Helicobacter pylori* CagL protein of the Cag type IV secretion system identifies novel functional domains

Tobias Bönig [1], Patrick Olbermann [1], Simon H. Bats[1], Wolfgang Fischer [2], Christine Josenhans[1*]

[1]Medizinische Hochschule Hannover, Institute for Medical Microbiology, Carl-Neuberg-Strasse 1, 30625 Hannover, Germany
[2]Ludwig Maximilian University Munich, Max-von-Pettenkofer Institute, Munich, Germany
[1,2]DZIF German Center of Infection Research, partner sites Hannover-Braunschweig and Munich

Supplementary Figures

Supplementary Figure S1: Alignment of CagL amino acid sequences derived from diveres CagL alleles of globally collected *H. pylori* strains belonging to different geographical populations.

Multiple sequence alignment of various CagL sequences from a global strain collection (Olbermann et al., 2009) was established for the identification of variable CagL sections and amino acids. The alignment was imaged by GeneDoc (Nicholas, K.B., Nicholas H.B. Jr., and Deerfield, D.W. II. 1997 GeneDoc: Analysis and Visualization of Genetic Variation, EMBNEW.NEWS 4:14), revealing several strain-specific CagL sequence variations. Identical stretches of sequence between all alleles are indicated in black with white letters. Variable amino acids are indicated with grey or white backgrounds. A consensus sequence was generated at the bottom of the alignment.

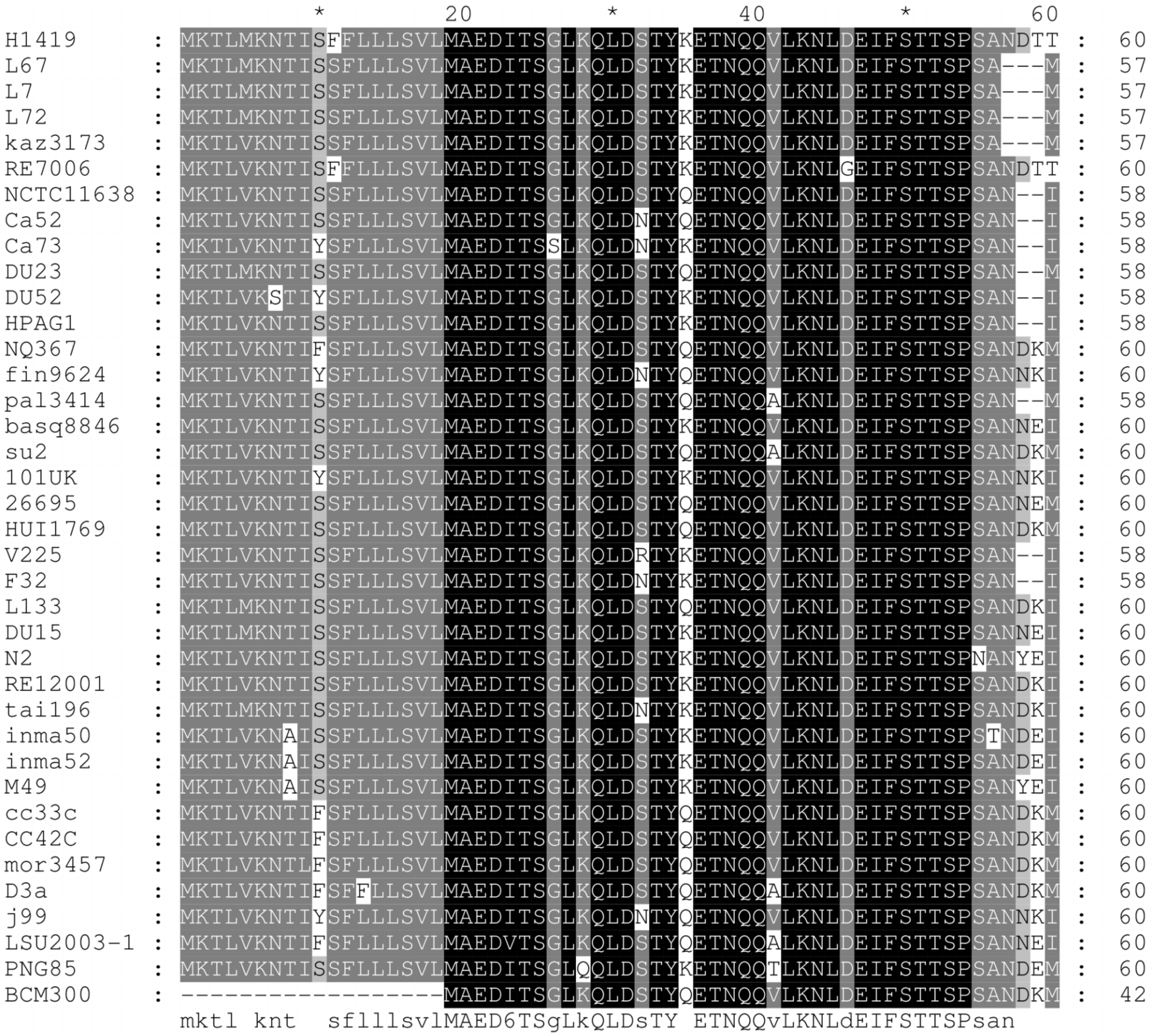

Supplementary Figure S1

```
                                    *        80         *       100         *       120
H1419       : GEEDALNIKKAAIALRGDLALLKANFEANELFFISEDVIFKTYMSNPELLLTYMKINPLD : 120
L67         : GEEDALNIKKAAIALRGDLALLKANFEANELFFISEDVIFKTYMSSPELLLTYMKINPLD : 118
L7          : GEEDALNIKKAAIALRGDLALLKANFEANELFFISEDVIFKTYMSSPELLLTYMKINPLD : 118
L72         : GEEDALNIKKAAIALRGDLALLKANFEANELFFISEDVIFKTYMSSPELLLTYMKINPLD : 118
kaz3173     : GEEDALNIKKAAIALRGDLALLKANFEANELFFISEDVIFKTYMSSPELLLTYMKINPLD : 118
RE7006      : GEEDALNIKKAAIALRGDLALLKANFEANELFFISEDVIFKTYMSSPELLLTYMKINPLD : 120
NCTC11638   : GQEDALNIKKAAIALRGDLALLKANFEANELFFISEDVIFKTYMSSPELLLTYMKINPLD : 118
Ca52        : GQEDALNIKKAAMALRGDLALLKANFEANELFFISEDVIFKTYMSSPELLLTYMKINPLD : 118
Ca73        : GQEDALNIKKAAIALRGDLALLKANFEANELFFISEDVIFKTYMSSPELLLTYMKINPLD : 118
DU23        : GEEDALNIKKAAIALRGDLALLKANFEANELFFISEDVIFKTYMSSPELLLTYMKINPLD : 118
DU52        : GQEDALNIKKAAIALRGDLALLKANFEANELFFISEDVIFKTYMSSPELLLTYMKINPLD : 118
HPAG1       : GQEDALNIKKAAIALRGDLALLKANFEANELFFISEDVIFKTYMSSPELLLTYMKINPLD : 118
NQ367       : GEEDALNIKKAAMALRGDLALLKANFEANELFFISEDVIFKTYMSSPELLLTYMKINPLD : 120
fin9624     : GQEDALNIKKAAIALRGDLALLKANFEANELFFISEDVIFKTYMSSPELLLTYMKINPLD : 120
pal3414     : GEEDALNIKKAAIALRGDLALLKANFEANELFFISEDVIFKTYMSSPELLLTYMKINPLD : 118
basq8846    : GQEDALNIKKAAIALRGDLALLKANFEANELFFISEDVIFKTYMSSPELLLTYMKINPLD : 120
su2         : GEEDALNIKKAAIALRGDLALLKANFEANELFFISEDVIFKTYMSSPELLLTYMKINPLD : 120
101UK       : GQEDALNIKKAAMALRGDLALLKANFEANELFFISEDVIFNTYMSSPELLLTYMKINPLD : 120
26695       : GEEDALNIKKAAIALRGDLALLKANFEANELFFISEDVIFKTYMSSPELLLTYMKINPLD : 120
HUI1769     : GEEDALNIKKAAIALRGDLALLKANFEANELFFISEDVIFKTYMSSPELLLTYMKINPLD : 120
V225        : GKEDALNIKKAAIALRGDLALLKANFEANELFFISEDVIFKTYMSSPELLLTYMKINPLD : 118
F32         : GQEDALNIKKAAIALRGDLALLKANFEANELFFISEDVIFKTYMSSPELLLTYMKINPLD : 118
L133        : GKEDALNIKKAAIALRGDLALLKANFEANELFFISEDVIFKTYMSSPELLLTYMKINPLD : 120
DU15        : GQEDALNIKKAAIALRGDLALLKANFEANELFFISEDVIFKTYMSSPELLLTYMKINPLD : 120
N2          : GKEDALNIKKAAIALKGDLALLKANFEANELFFISEDVIFKTYMSSPELLLTYMKINPLD : 120
RE12001     : GKEDALNIKKAAIALRGDLALLKANFEANELFFISEDVIFKTYMSSPELLLTYMKINPLD : 120
tai196      : GKEDALNIKKAAIALRGDLALLKANFEANELFFISEDVIFKTYMSsPELLLTYMKINPLD : 120
inma50      : GKEDALNIKKAAIALKGDLALLKANFEANELFFISEDVIFKTYMSSPELLLTYMKINPLD : 120
inma52      : GKEDALNIKKAAIALKGDLALLKANFEANELFFISEDVMFKTYMSSPELLLTYMKINPLD : 120
M49         : GKEDALNIKKAAIALRGDLALLKANFEANELFFISEDVIFKTYMSSPELLLTYMKINPLD : 120
cc33c       : GEEDALNIKKAAMALRGDLALLKANFEANELFFISEDVIFKTYMSSPELLLTYMKINPLD : 120
CC42C       : GEEDALNIKKAAIALRGDLALLKANFEANELFFISEDVIFKTYMSSPELLLTYMKINPLD : 120
mor3457     : GEEDALNIKKAAMALRGDLALLKANFEANELFFISEDVIFKTYMSSPELLLTYMKINPLD : 120
D3a         : GEEDALNIKKAAMALRGDLALLKANFEANELFFISEDVIFKTYMSSPELLLTYMKINPLD : 120
j99         : GQEDALNIKKAAIALRGDLALLKANFEANELFFISEDVIFKTYMSSPELLLTYMKINPLD : 120
LSU2003-1   : GQEDALNIKKAAIALRGDLALLKANFEANELFFISEDVIFKTYMSSPELLLTYMKINPLD : 120
PNG85       : GKEDALNIKKAAIALRGDLALLKANFEANELFFISEDVIFKTYMSSPELLLIYMKINPLD : 120
BCM300      : GEEDALNIKKAAMALRGDLALLKANFEANELFFISEDVIFKTYMSSPELLLTYMKINPLD : 102
              G EDALNIKKAA6AL4GDLALLKANFEANELFFISEDV6FkTYMSsPELLLtYMKINPLD
```

Supplementary Figure S1

```
                                *         140         *         160         *         180
H1419       : QNTAEQQCGISDKVLVLYCEGKLKIEQEKQNIRERLETSLKAYQSNIGGTTSLITASQTL : 180
L67         : QNTAEQQCGISDKVLVLYCEGKLKIEQEKQNIRERLETSLKAYQSNIGGTTSLITASQTL : 178
L7          : QNTAEQQCGISDKVLVLYCEGKLKIEQEKQNIRERLETSLKAYQSNIGGTASLITASQTL : 178
L72         : QKTAEQQCGISDKVLVLYCEGKLKIEQEKQNIRERLETSLKAYQSNIGGTASLITASQTL : 178
kaz3173     : QNTAEQQCGISDKVLVLYCEGKLKIEQEKQNIRERLETSLKAYQSNIGGTASLITASQTL : 178
RE7006      : QNTAEQQCGISDKVLVLYCEGKLKIEQEKQNIRERLETSLKAYQSNIGGTTSLITASQTL : 180
NCTC11638   : QNTAEQQCGISDKVLVLYCEGKLKIEQEKQNIRERLETSLKAYQSNIGGTASLITASQTL : 178
Ca52        : QNTAEQQCGISDKVLVLYCEGKLKIEQEKQNIRERLETSLKAYQSNIGGTTSLITASQTL : 178
Ca73        : QNTAEQQCGISDKVLVLYCEGKLKIEQEKQNIRERLETSLKAYQSNIGGTASLITASQTL : 178
DU23        : QNTAEQQCGISDKVLVLYCEGKLKIEQEKQNIRERLETSLKAYQSNIGGAASLITASQTL : 178
DU52        : QNTAEQQCGISDKVLVLYCEGKLKIEQEKQNIRERLETSLKAYQSNIGGTASLITASQTL : 178
HPAG1       : QNTAEQQCGISDKVLVLYCEGKLKIEQEKQNIRERLETSLKAYQSNIGGTASLITASQTL : 178
NQ367       : QKTAEQQCGISDKILVLYCGGKLKIEQEKQNIRERLETSLKAYQSNIGGTASLIIASQTL : 180
fin9624     : QNTAEQQCGISDKVLVLYCEGKLKIEQEKQNIRERLETSLKAYQSNIGGTASLITASQTL : 180
pal3414     : QKTAEQQCGISDKILVLYCEGKLKIEQEKQNIRERLETSLKAYQSNIGGTASLITASQTL : 178
basq8846    : QNTAEQQCGISDKVLVLYCEGKLKIEQEKQNIRERLETSLKAYQSNIGGTASLITASQTL : 180
su2         : QKTAEQQCGISDKILVLYCWGKLKIEQEKQNIRERLETSLKAYQSNIGGTASLIIASQTL : 180
101UK       : QNTAEQQCGIFDKVLVLYCEGKLKIEQEKQNIRERLETSLKAYQSNIGGTASLITASQTL : 180
26695       : QNTAEQQCGISDKVLVLYCEGKLKIEQEKQNIRERLETSLKAYQSNIGGTASLITASQTL : 180
HUI1769     : QKTAEQQCGISDKILVLYCEGKLKIEQEKQNIRERLETSLKAYQSNIGGTASLIIASQTL : 180
V225        : QNTAEQQCGISDKVLVLYCEGKLKIEQEKQNIRERLETSLKAYQSNIGGTASLITASQTL : 178
F32         : QNTAEQQCGISDKVLVLYCEGKLKIEQEKQNIRERLETSLKAYQSNIGGTASLITASQTL : 178
L133        : QKTAEQQCGISDKVLVLYCEGKLKIEQEKQNIRERLETSLKAYQSNIGGTTSLITASQTL : 180
DU15        : QNTAEQQCGISDKVLVLYCEGKLKIEQEKQNIRERLETSLKAYQSNIGGTTSLITASQTL : 180
N2          : QNTAEQQCGISDKVLVLYCEGKLKIEQEKQNIRERLETSLKAYQSNIGGTASLITASQTL : 180
RE12001     : QKTAEQQCGISDKVLVLYCEGKLKIEQEKQNIRERLETSLKAYQSNIGGTASLITASQTL : 180
tai196      : QKTAEQQCGISDKVLVLYCEGKLKIEQEKQNIRERLETSLKAYQSNIGGTASLITASQTL : 180
inma50      : QETTEQQCGISDKVLVLYCGGKLKIEQEKQNIRERLETSLKAYQSNIGGTVSLITASQTL : 180
inma52      : QETTDQQCGISDKVLVLYCEGKLKIEQEKQNIRERLETSLKAYQSNIGGTASLITASQTL : 180
M49         : QETTEQQCGISDKVLVLYCGGKLKIEQEKQNIRERLETSLKAYQSNIGGTASLITASQTL : 180
cc33c       : QKTAEQQCGISDKILVLYCEGKLKIEQEKQNIRERLETSLKAYQSNIGGTASLITASQTL : 180
CC42C       : QKTAEQQCGISDKILVLYCEGKLKIEQEKQNIRERLETSLKAYQGNIGGTASLITASQTL : 180
mor3457     : QKTAEQQCGISDKILVLYCEGKLKIEQEKQNIRERLETSLKAYQSNIGGTASLITASQTL : 180
D3a         : QKTAEQQCGISDKILVLYCEGKLKIEQEKQNIRERLETSLKAYQSNIGGTASLIIASQTL : 180
j99         : QKTAEQQCGISDKVLVLYCEGKLKIEQEKQNIRERLETSLKAYQSNIGGTASLITASQTL : 180
LSU2003-1   : QNTAEQQCGISDKVLVLYCEGKLKIEQEKQNIRERLETSLKAYQSNIGGTASLITASQTL : 180
PNG85       : QKTAEQQCGISDKVLVLYCGGKLKIEQEKQNIRERLETSLKAYQSNIGGTASLITASQTL : 180
BCM300      : QKTAEQQCGISDKILVLYCEGKLKIEQEKQNIRERLETSLKAYQSNIGGTASLIIASQTL : 162
              Q TaeQQCGIsDK6LVLYCeGKLKIEQEKQNIRERLETSLKAYQsNIGGtaSLItASQTL
```

Supplementary Figure S1

```
                     *        200        *        220        *
H1419     : VESLKNKNFIKGIRKLMLAHNKVFLNYLEKLDALERSLEQSKRQYLQERQSSKIIVK- : 237
L67       : VESLKNKNFIKGIRKLMLAHNKVFLNYLEKLDALEISLEQSKRQYLQERQSSKIIVK- : 235
L7        : VESLKNKNFIKGIRKLMLAHNKVFLNYLEKLDALEISLEQSKRQYLQERQSSKIIVK- : 235
L72       : VESLKNKNFIKGIRKLMLAHNKVFLNYLEELDALERSLEQSKRQYLQERQSSKIIVK- : 235
kaz3173   : VESLKNKNFIKGIRKLMLAHNKVFLNYLEELDALERSLEQSKRQYLQERQSSKIIVK- : 235
RE7006    : VESLKNKNFIKGIRKLMLAHNKVFLNYLEKLDALERSLEQSKRQYLQERQSSKIIVK- : 237
NCTC11638 : VESLKNKNFIKGIRKLMLAQNKVFLNYLEELDALERSLEQSKRQYLQERQSSKIIVK- : 235
Ca52      : VESLKNKNFIKGIRKLMLAHNKVFLNYLEELDALERSLEQSKRQYLQERQSSKIIVK- : 235
Ca73      : VESLKNKNFIKGIRKLMLAHNKIFLNYLEELDALERSLEQSKRQYLQERQSSKIIVK- : 235
DU23      : VESLKNKNFIKGIRKLMLAHNKVFLNYLEELDALERSLEQSKRQYLQERQSSKIIVK- : 235
DU52      : VESLKNKNFIKGIRKLMLAHNKVFLNYLEELDALERSLEQSKRQYLQERQSSKIIVK- : 235
HPAG1     : VESLKNKNFIKGIRKLMLAHNKVFLNYLEELDALERSLEQSKRQYLQERQSSKIIVK- : 235
NQ367     : VESLKNKNFIKGIRKLMLAQNKVFLNYLEELDALERSLEQSKRQYLQERQSSKIIVK- : 237
fin9624   : VESLKNKNFIKGIRKLMLAHNKVFLNYLEELDALESSLEQSKRQYLQERQSSKIIVK- : 237
pal3414   : VESLKNKNFIKGIRKLMLAQNKVFLNYLEELDALERSLEQSKRQYLQERQSSKIIVK- : 235
basq8846  : VESLKNKNFIKGIRKLMLAHNKVFLNYLEELDALERSLEQSKRQYLQERQSSKIIVK- : 237
su2       : VESLKNKNFIKGIRKLMLAHNKVFLNYLEELDALERSLEQSKRQYLQERQSSKIIVK- : 237
101UK     : VESLKNKNFIKGIRKLMLAHNKVFLNYLEKLDALERSLEQSKRQYLQERQSSKIIVK- : 237
26695     : VESLKNKNFIKGIRKLMLAHNKVFLNYLEELDALERSLEQSKRQYLQERQSSKIIVK- : 237
HUI1769   : VESLKNKNFIKGIRKLMLAHNKVFLNYLEELDALERSLEQNKRQYLQERQSSKIIVK- : 237
V225      : VESLKNKNFIKGIRKLMLAHNKVFLNYLEKLDALEISLEQSKRQYLQERQSSKIIVK- : 235
F32       : VESLKNKNFIKGIRKLMLAHDKVFLNYLEKLDALEISLEQSKRQYLQERQSSKVIVK- : 235
L133      : VESLKNKNFIKGIRKLMLAHNKVFLNYLEELDALEISLEQSKRQYLQERQSSKVIVK- : 237
DU15      : VESLKNKNFIKGIRKLMLAHDKVFLNYLEKLDALEISLEQSKRQYLQERQSSKVIVK- : 237
N2        : VESLKNKNFIKGIRKLMLAHDKVFLNYLEKLDALEISLEQSKRQYLQERQSSKVIVK- : 237
RE12001   : VESLKNKNFIKGIRKLMLAHDKVFLNYLEKLDALEISLEQNKRQYLQERQSSKVIVK- : 237
tai196    : VESLKNKNFIKGIRKLMLAHDKVFLNYLEKLDALEISLEQSKRQYLQERQSSKVIVK- : 237
inma50    : VESLKNKNFIKGIRKLMLAHNKVFLNYLEKLDALEISLEQSKRQYLQERQSSKIIVK- : 237
inma52    : VESLKNKNFIKGIRKLMLAHNKVFLNYLEKLDALEISLEQNKRQYLQERQSSKIIVK- : 237
M49       : VESLKNKNFIKGIRKLMLAHNKVFLNYLEKLDALEISLEQSKRQYLQERQSSKIIVK- : 237
cc33c     : VESLKNKNFIKGIRKLMLAHNKVFLNYLEELDALERSLEQSKRQYLQERQSSKIIVK- : 237
CC42C     : VESLKNKNFIKGIRKLMLAQNKVFLNYLEELDALERSLEQSKRQYLQERQSSKIIVK- : 237
mor3457   : VESLKNKNFIKGIRKLMLAHNKVFLNYLEELDALERSLEQSKRQYLQERQSSKIIVK- : 237
D3a       : VESLKNKNFIKGIRKLMLAHNKVFLNYLEELDALERSLEQSKRQYLQERQSSKIIVK- : 237
j99       : VESLKNKNFIKGIKKLMLAHNKVFLNYLEELDALERSLEQSKRQYLQERQSSKIIVK- : 237
LSU2003-1 : VESLKNKNFIKGIRKLMLAHNKVFLNYLEELDALERSLEQSKRQYLQERQSSKIIVK- : 237
PNG85     : VESLKNKNFIKGIRKLMLAHNKVFLNYLEKLDALERSLEQSKWQYLQERQSSKIIVK- : 237
BCM300    : VESLKNKNFIKGIRKLMLAHNKVFLNYLEELDALERSLEQSKRQYLQERQSSKIIVK- : 219
            VESLKNKNFIKGI4KLMLAh1K6FLNYLE LDALE SLEQsKrQYLQERQSSK6IVK
```

Supplementary Figure S1

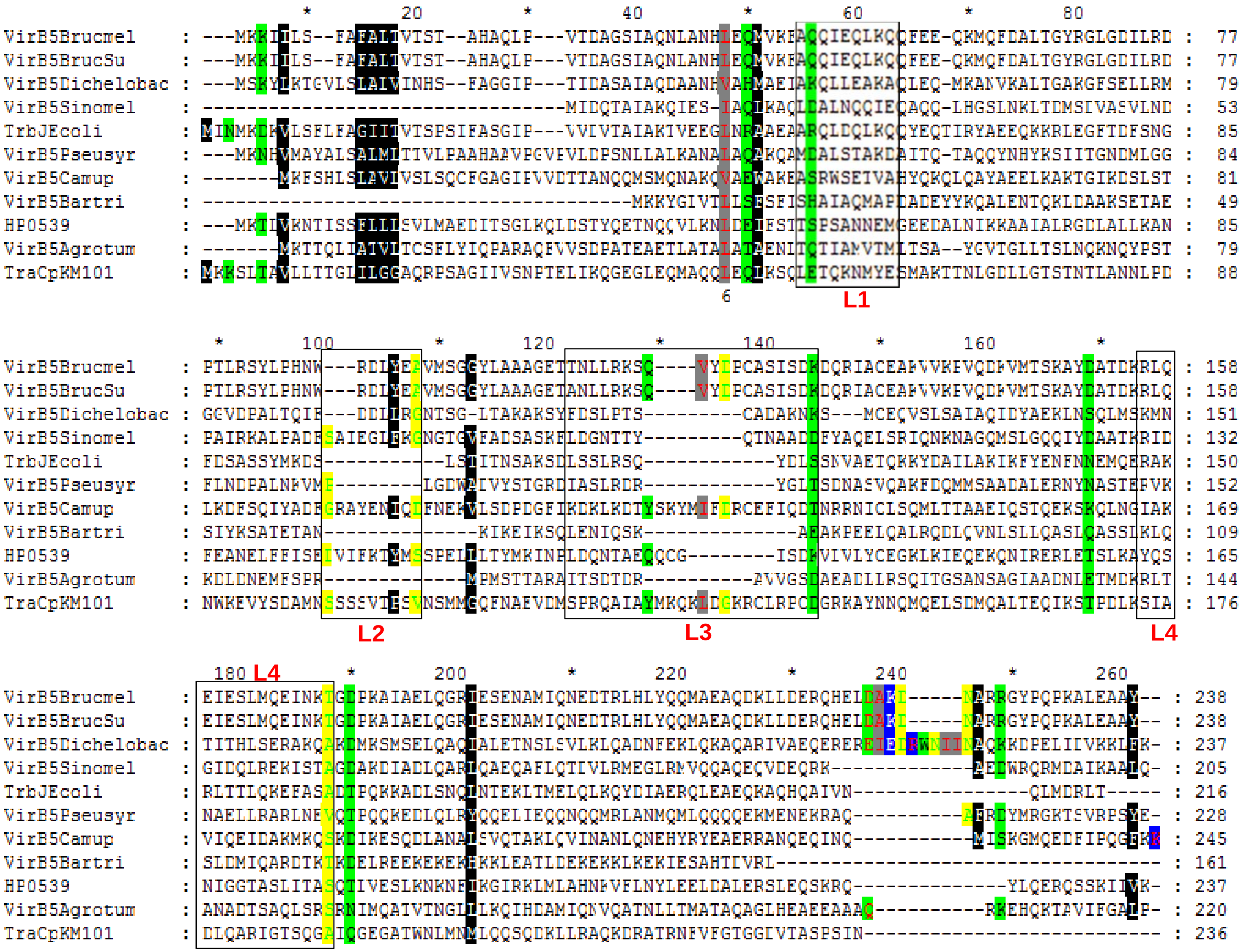

Supplementary Figure S2: amino acid alignment of putative T4SS tip proteins (VirB5 orthologs) from diverse bacteria, including HP0539 (CagL) from *H. pylori* strain 26695.
The alignment was generated by CLUSTAL-W and manually curated. The alignment is presented using GeneDoc (Nicholas, K.B., Nicholas H.B. Jr., and Deerfield, D.W. II. 1997 GeneDoc: Analysis and Visualization of Genetic Variation, EMBNEW.NEWS 4:14), with amino acids of similar physicochemical properties shaded in the same color* by software default settings. *H. pylori*–specific amino acid motifs (in predicted loops which are either common to all orthologs or specific to certain host-associated species) are boxed (loop L1 to loop L4 labelled in red). TrbJ and TraC are VirB5 orthologs from DNA-transporting T4SS; Proteins designated VirB5 are recognized, annotated T4SS VirB5 proteins in databases.
Bacterial names are abbreviated as follows: Brucmel: *Brucella melitensis*; Brucsu: *Brucella suis*; Dichelobac: *Dichelobacter nodosum*; Sinomel: *Sinorhizobium meliloti*; Ecoli: *Escherichia coli*; Pseusyr: *Pseudomonas syringae*; Camup: *Campylobacter upsaliensis*; Bartri: *Bartonella tribocorum*; Agrotum: *Agrobacterium tumefaciens.*
*The color coding according to software default settings is as follows: 1) Black background color (white text color): hydrophobic amino acids (ILVAGMFYWHPTC); 2) Green background color (blue text color): polar amino acids (YWHKREQDNST); 3) Blue background color (white text color): charged amino acids (HKRED); 4) Grey background color (red text color): aliphatic amino acids (ILVA); 5) Yellow background color (green text color): small amino acids (VCAGDNSTP); 6) Green background color (red text color): amphoteric amino acids (HREQDN); 7) Blue background color (red text color): amino acids with possible positive charge (HKR).

Supplementary Figure S2

```
HP0539: MKTLVKNTISSFLLLSVLMAEDITSGLKQLDSTYQETNQQVLKNLDEIFSTTSPSANNEM
Jpred:  -HHHHHHHHHHHHHHHHHHHHHHHHHHHHHH---HHHHHHHHHHHHHHHH----------
Konf.:  709998610478888888860189999874000088869999998877514777777777

HP0539: GEEDALNIKKAAIALRGDLALLKANFEANELFFISEDVIFKTYMSSPELLLTYMKINPLD
Jpred:  ------HHHHHHHHE---EEEEE-------EEEE---EEEEH------HEEEEEE-----
Konf.:  777653000000000055000001536873688704400000047800000088605777

HP0539: QNTAEQQCGISDKVLVLYCEGKLKIEQEKQNIRERLETSLKAYQSNIGGTASLITASQTL
Jpred:  ------------EEEEEEE---EEEHHHHHHHHHHHHHHHHH---------HHHH-HHHH
Konf.:  766443467775278998458556020668999999757764005778760110000678

HP0539: VESLKNKNFIKGIRKLMLAHNKVFLNYLEELDALERSLEQSKRQYLQERQSSKIIVK
Jpred:  HHHH----HHHHHHHHHHH--HHHHHHHHHHHHHHHHHHHHHHHHHHHH---EEEE-
Konf.:  752068866899999873008167652265042288757759999998038806736
```

deletion

Supplementary Figure S3: Secondary structure prediction of *H. pylori* CagL (strain 26695); indicated is the selection of ten short motif deletion mutants.
Secondary structural prediction of CagL was performed using Jpred (www.compbio.dundee.ac.uk/jpred).
Helical (H), extended (E) and other (-) types of secondary structure. Confidence of prediction: (9) high, (0) low.

Supplementary Figure S3

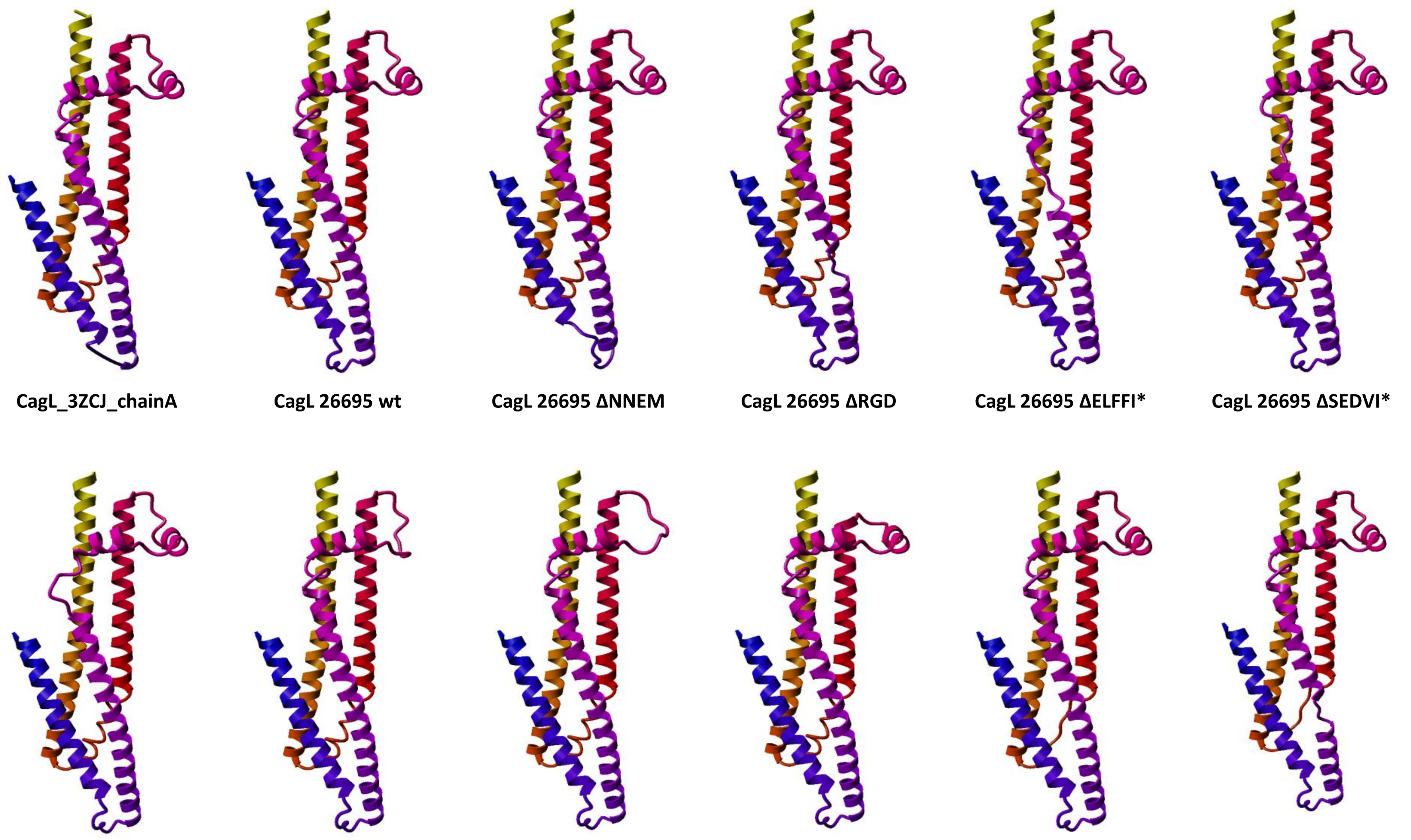

CagL_3ZCJ_chainA | CagL 26695 wt | CagL 26695 ΔNNEM | CagL 26695 ΔRGD | CagL 26695 ΔELFFI* | CagL 26695 ΔSEDVI*

CagL 26695 ΔFKTYM* | CagL 26695 ΔLDQNT* | CagL 26695 ΔAEQQC | CagL 26695 ΔGISDK* | CagL 26695 ΔTASLI | CagL 26695 ΔRGD ΔTASLI

Supplementary Figure S4: Template-based structural prediction of different CagL site-directed motif deletion mutants based on the published CagL crystal structure (pdb 3ZCJ_chainA; Barden *et al.*, Structure 2013). Template-based structural prediction of different CagL deletion mutants using the fully automated protein structure homology-modelling server SwissModel (Biasini M. *et al.*, Nucleic Acids Research 2014). The CagL crystal structure (pdb: 3ZCJ_chainA; Barden S. *et al.*, Structure 2013) shown for comparison was imaged in ribbon mode by Yasara (www.yasara.org). Orientation: α 90.739; β 100.239; γ 25.757. The template-based structural prediction does not allow a conclusion regarding the integrity and stability of the native proteins in *H. pylori* (see also Fig. 2 in main manuscript: CagL variants with hampered integrity *in vivo* according to Fig. 2 are marked by *).

Supplementary Figure S4

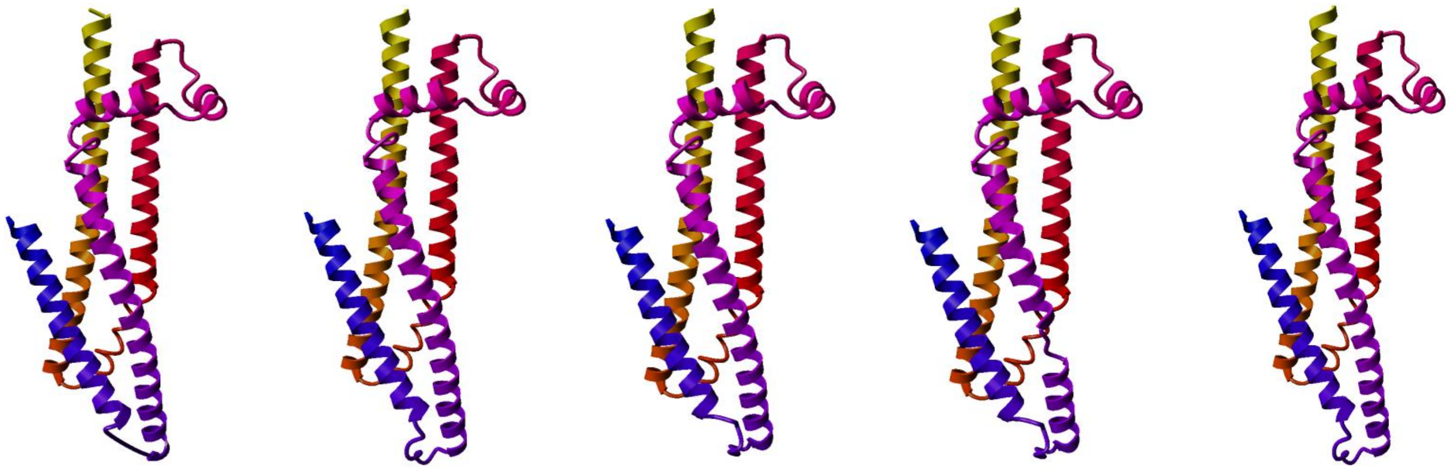

CagL_3ZCJ_chainA | CagL 26695 wt | CagL 26695 ΔTSPSA | CagL 26695 ΔTSPSA ΔRGD | CagL 26695 T170K

Orientation
α 90.739
β 100.239
γ 25.757

Supplementary Figure S4

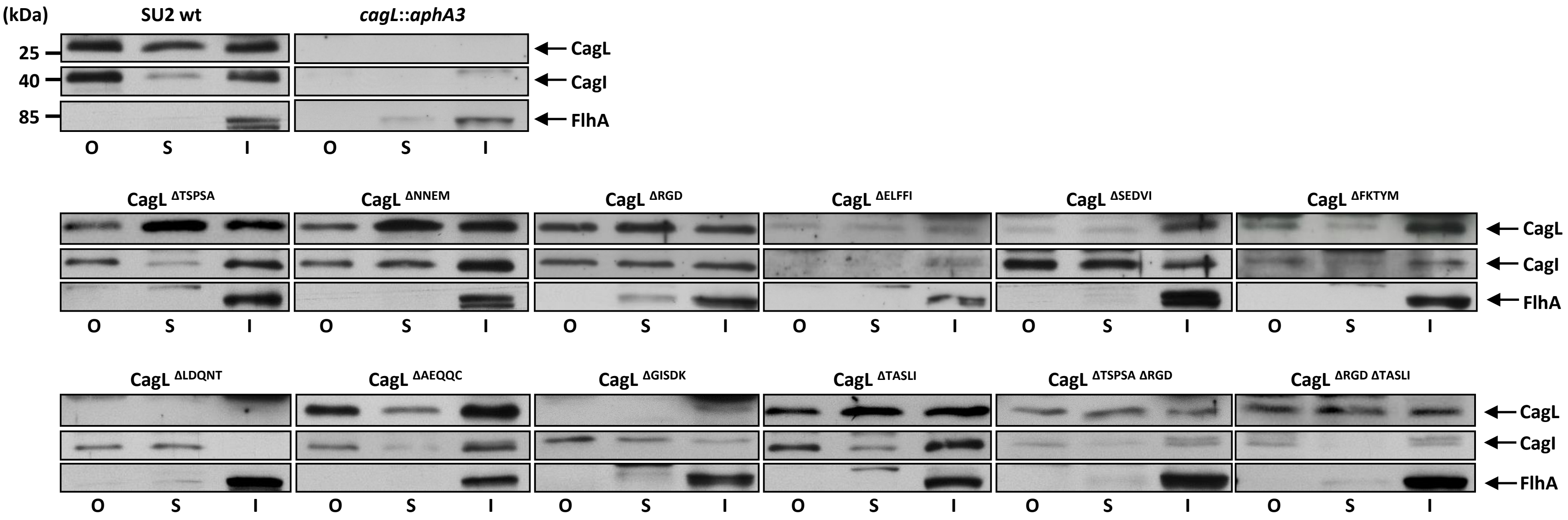

Supplementary Figure S5: Subcellular localization of CagL and CagI in several isogenic chromosomal mutants of *H. pylori* SU2 containing site-directed motif deletions in *cagL*.

Bacteria were separated into outer (O), soluble (S) and insoluble (I) fractions by shearing, sonication and differential centrifugation (Methods). Equal protein amounts (10 µg) for each sample were separated on SDS gels followed by Western blotting. Proteins (CagL, CagI and FlhA) were detected using specific antisera (rabbit α-CagL, 1:20,000; rabbit α-CagI, 1:5,000; rabbit α-FlhA, 1:10,000). FlhA (membrane-bound component of the flagellar T3 export apparatus) served as a fractionation control of the insoluble fraction (IM). Representative blots of one mutant clone for each CagL mutation (two clones each were characterized with equivalent results) are shown.

Supplementary Figure S5

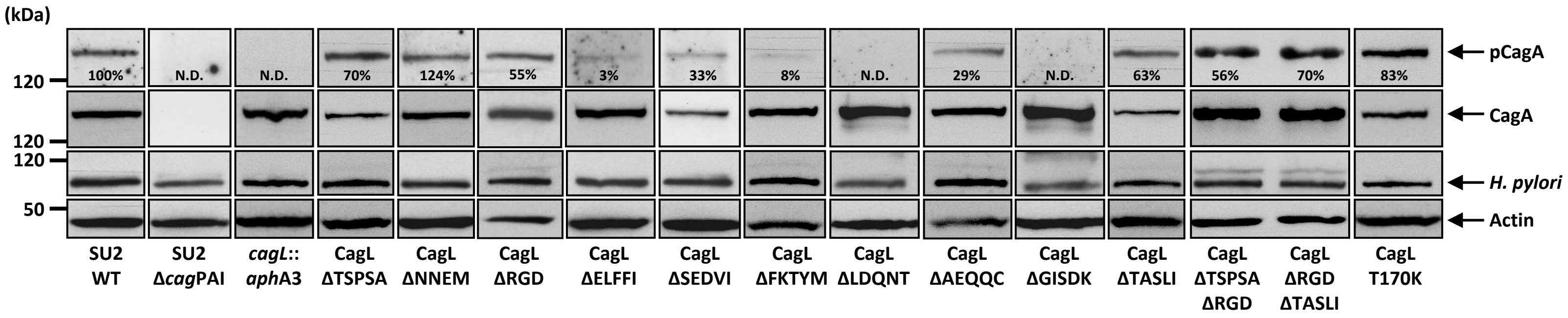

Supplementary Figure S6: CagA translocation assay into AGS cells for different CagL mutants, showing the contribution of different CagL motifs to the translocation of CagA into AGS cells.

AGS cells were infected for 4 h (MOI=100) with wild-type SU2 and isogenic chromosomal *cagL* deletion and substitution mutants
as indicated in the figure. AGS cell lysates were analyzed for phosphorylated CagA (pCagA; rabbit anti-*Hp*-pCagA, CSPEPI-pY-ATID, IgG-fraction, 1:10,000) and total CagA (CagA; rabbit α-*Hp*-CagA-antigen, IgG-fraction, Austral Biologicals, San Ramon, USA, 1:10,000). Detection of invariable *H. pylori* antigens (*H. pylori*; rabbit anti-*H. pylori*, DAKO, 1:2,500) for *H. pylori* quantitation and the cellular protein actin (Actin; mouse α-actin, Millipore, Schwalbach, Germany, 1:20,000). were used as loading controls. The influence of the respective *cagL* mutations on pCagA and CagA protein amounts was determined by Western blot densitometry (normalization against wild type samples and the invariable *H. pylori* antigens was done in each respective blot separately). Phospho-CagA signal for *H. pylori* SU2 parental strain of each blot was normalized against *H. pylori* invariable antigen intensity and then set to 100% to serve as intensity standard separately for each blot. The relative values for pCagA in the mutants are indicated in percent (black labels in the pCagA panels). Representative panels of one mutant clone for each CagL mutation (two clones each were characterized with equivalent results) are shown. N.D.= not detectable.

Supplementary Figure S6

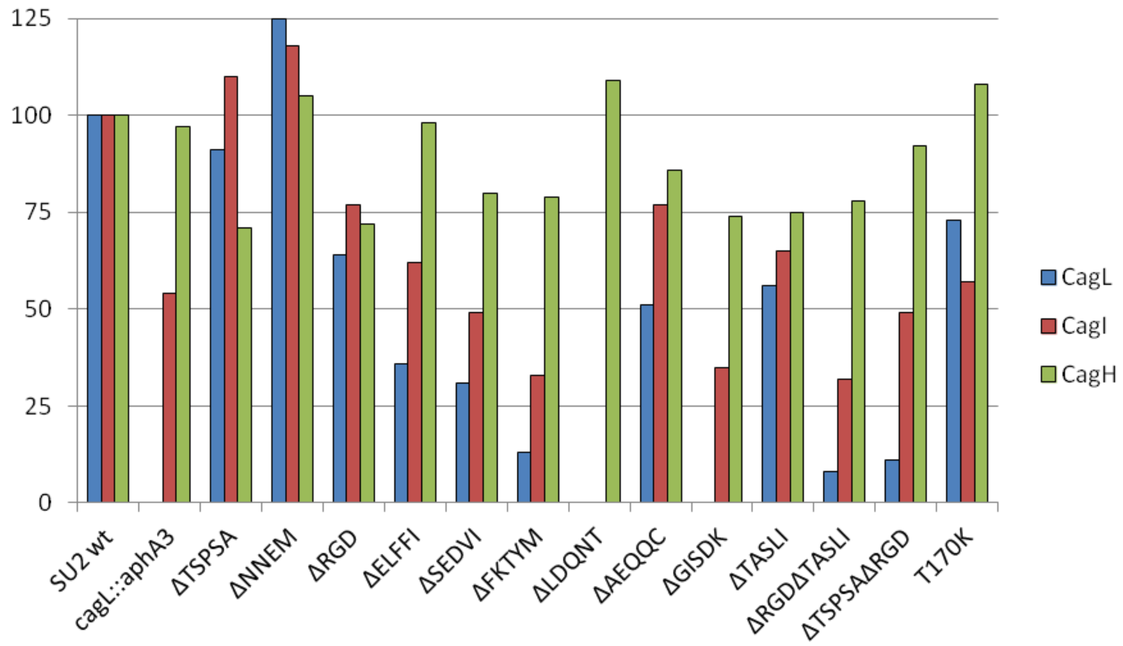

Supplementary Figure S7: Quantitation (densitometry) of CagL, CagI and CagH in several isogenic chromosomal single amino acid substitution mutants of *H. pylori* SU2 in *cagL*.

Equal protein amounts of bacterial total lysates (10 µg) were separated on SDS gels followed by Western blotting. Proteins (CagL, CagI and CagH) were detected using specific antisera (rabbit α-CagL, 1:20,000; rabbit α-CagI, 1:5,000; rabbit α-CagH, 1:5,000). Densitometry was performed using ImageJ on the Western blots. Intensities for each protein band were corrected for equal loading (using one defined invariable *H. pylori* antigen band for each sample) and then normalized against the respective CagL, CagI or CagH protein band intensities in a wild type sample (reference) run on each blot, which were set to 100%. Results are shown in this graph as relative intensities in comparison to the wild type sample. The quantitation refers to the panel shown in main Fig. 2.

Supplementary Figure S7

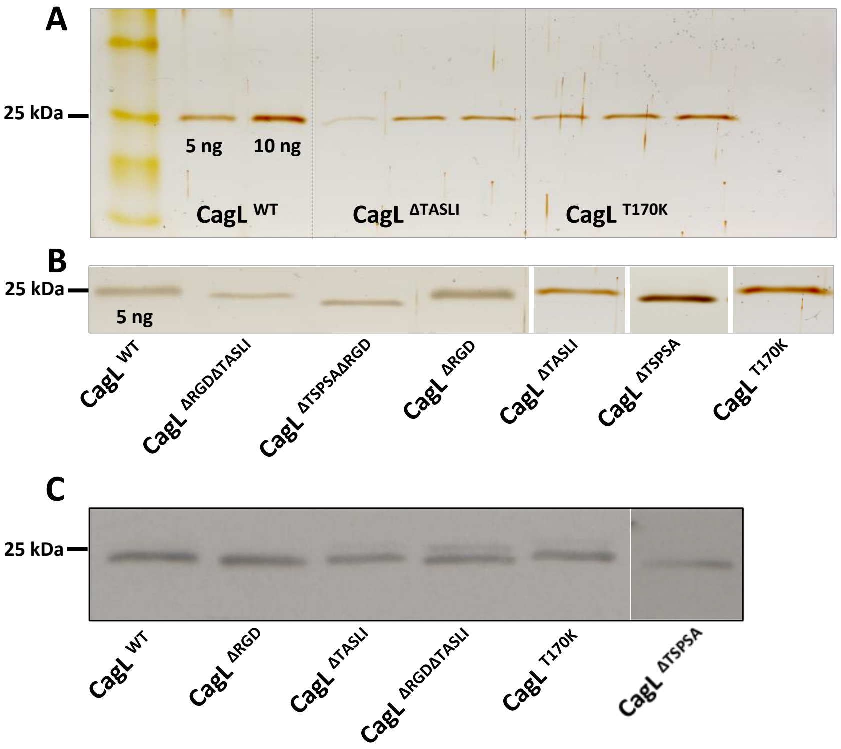

Supplementary Fig. S8: Silver stains and Western blots of analyzed purified CagL protein variants for purity control and comparative quantification.

Non-tagged CagL proteins after two-step purification were separated on SDS-gels followed by silver staining (Blum H. *et al.*, Electrophoresis 1987). Protein amounts were always precisely assessed by direct comparison with defined GST amounts (control protein) and the amounts adjusted for the subsequent binding assays by comparison with several different lanes loaded with defined CagL wild type protein amounts on each gel. Panel **A)** shows an exemplary gel with CagL wild type protein (reference amounts) and two purified mutant proteins loaded for comparison in different amounts, respectively. Panel **B)** shows silver-stained gel results of all purified CagL variants used in this study. Panel **C)** shows a Western blot after loading defined amounts of CagL variants (25 ng in each lane), and detected using anti-CagL polyclonal antiserum (AK271, 1:10,000; Methods). This was used as an additional control to verify that all variants were detected at similar detection intensities by the antiserum. CagL$^{\Delta TSPSA\Delta RGD}$ was only tested in ELISA (results not shown)

Supplementary Figure S8

A

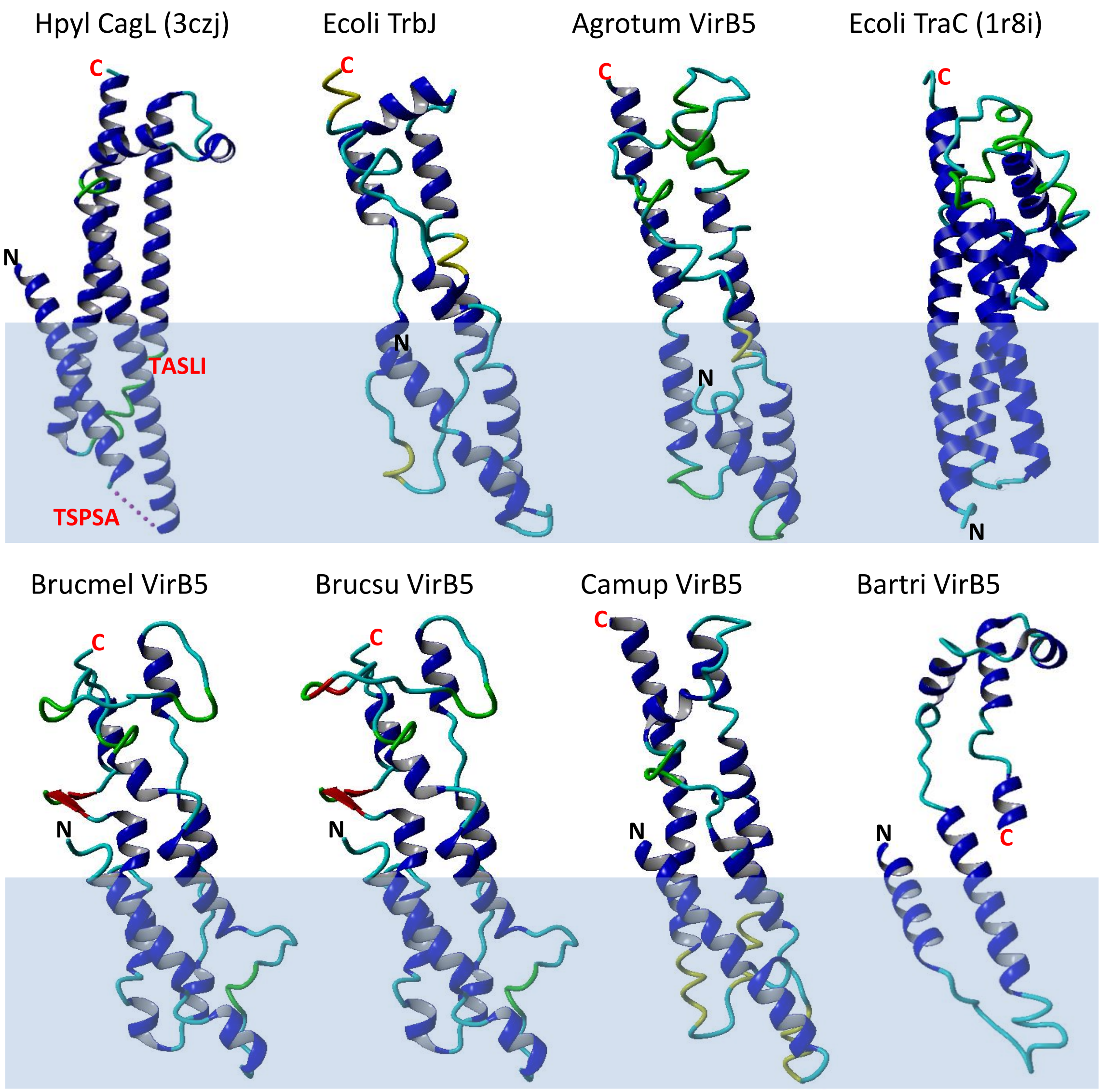

B

	Helix	Sheet	Turn	Coil	3/10 Helix
Hpyl CagL 3zcj	87.8	0	7.8	4.4	0
Ecoli TrbJ	56.6	0	2.6	38.2	2.6
Agrotum VirB5	53.4	0	9.0	35.4	2.2
Ecoli TraC 1r8i	63.6	0	8.6	25.1	2.7
Brucmel VirB5	49.7	2.6	9.5	38.1	0
Brucsu VirB5	49.7	2.6	5.3	42.3	0
Camup VirB5	61.7	0	4.4	22.2	11.7
Bartri VirB5	85.3	0	0	14.7	0
average	65.6	0.7	2.6	38.2	2.6

Supplementary Fig. S9:

A) CagL orthologous VirB5 proteins from different bacterial species were modelled using SwissModel according to the crystal structure of CagL chainA (pdb: 3zcj; Barden *et al*., Structure 2013). *Escherichia coli* TraC, a VirB5 ortholog from a plasmid conjugation system, which provided the first published VirB5 structure (pdb: 1r8i; Yeo *et al*., PNAS 2003), is shown for comparative purposes. Modelling scores (QMEAN4) were at -6 or lower for each protein, indicating an uncertain structural model (not all amino acids were modelled in each ortholog).

Short designation of proteins and bacterial species above the protein structure follows the designations in Supplementary Figure S1: Hpyl = *H. pylori*; Ecoli = *E. coli*; Agrotum = *A. tumefaciens*; Brucmel = *B. melitensis*; Brucsu = *B. suis*; Camup = *C. upsaliensis*; Bartri = *B. tribocorum*.

Structures were visualized and images extracted in the Yasara software; image extraction of each ortholog was performed from superposed structures. N- termini (N, black letter) and C-termini (C, red letter) are arranged in the same orientation as in the superposition (indicated in each structure). The lower parts of the proteins containing disordered regions/loops with the potential to interact with a target bacterial or host cell (according to the TSPSA and TASLI loops within CagL – indicated in red) are boxed in light blue. **B)** Same proteins as in **A)** were analyzed for predicted content in secondary structure using the Yasara software.

blue: a-helices
red: b-sheets
green/turquoise: loop, disordered region
purple: not resolved in crystal structure.

Supplementary Figure S9

Supplementary Tables

Supplementary Table 1: Bacterial strains

***H. pylori* strains**	**Comment**	**Reference**
26695	Wild type strain (hpEurope)	(1)
SU2	Wild type strain (hpNEAfrica)	(2)
SU2 ΔCagL (Km)	SU2 *cagL*::*aphA3´*-III	This study
SU2 ΔCagL (Km-sacB)	SU2 *cagL*::*aphA3´*-III-*sacB*	This study
SU2 ΔCagL (Km) ΔCagI (Cm)	SU2 *cagL*::*aphA3´*-III *cagI*::*cat*	This study
SU2 ΔCagL (Km) ΔCagH (Cm)	SU2 *cagL*::*aphA3´*-III *cagH*::*cat*	This study
SU2 $CagL^{\Delta TSPSA}$	Chromosomal deletion of TSPSA codons in *cagL*	This study
SU2 $CagL^{\Delta NNEM}$	Chromosomal deletion of NNEM codons in *cagL*	This study
SU2 $CagL^{\Delta SEDVI}$	Chromosomal deletion of SEDVI codons in *cagL*	This study
SU2 $CagL^{\Delta FKTYM}$	Chromosomal deletion of FKTYM codons in *cagL*	This study
SU2 $CagL^{\Delta GISDK}$	Chromosomal deletion of GISDK codons in *cagL*	This study
SU2 $CagL^{\Delta LDQNT}$	Chromosomal deletion of LDQNT codons in *cagL*	This study
SU2 $CagL^{\Delta RGD}$	Chromosomal deletion of RGD codons in *cagL*	This study
SU2 $CagL^{\Delta ELFFI}$	Chromosomal deletion of ELFFI codons in *cagL*	This study
SU2 $CagL^{\Delta AEQQC}$	Chromosomal deletion of AEQQC codons in *cagL*	This study
SU2 $CagL^{\Delta TASLI}$	Chromosomal deletion of TASLI codons in *cagL*	This study
SU2 $CagL^{T170K}$	Chromosomal substitution of T170K codon in *cagL*	This study
SU2 $CagL^{\Delta TSPSA\ \Delta RGD}$	Chromosomal double deletion of TSPSA and RGD codons in *cagL*	This study
SU2 $CagL^{\Delta RGD\ \Delta TASLI}$	Chromosomal double deletion of RGD and TASLI codons in *cagL*	This study

***E. coli* strains**	**Genotype**	**Reference**
DH5α	F^- *endA1 recA1 hsdR17* Δ(*lacZYA-argF*) *U169 thi1 supE44 gyrA96 relA1*	(3)
MC1061	F^- *araD139* Δ(*ara-leu*) *7696 galE15 galK16* Δ(lac)*X74 rpsL* (*Str*^{r}) *hsdR2* (r_K^+ m_K^+) *mcrA mcrB1*	(4)
XL1-Blue	*recA1 endA1 gyrA96 thi-1 hsdR17 supE44 relA1 lac* [F´ *proAB lacI*^{q}*Z*Δ*M15* Tn*10* (Tet^r)]	Stratagene
XL10-Gold	Tet^{r}Δ(*mcrA*)*183* Δ(*mcrCB-hsdSMR-mrr*)*173 endA1 supE44 thi-1 recA1 gyrA96 relA1 lac Hte* [F´ *proAB lacI*^{q}*Z*Δ*M15* Tn*10* (Tet^r) Amy Cam^r]	Stratagene
BL21 (DE3)	F^- *dcm ompT hsdS* (r_B^- m_B^-) *gal* λ (DE3)	(5)

Supplementary Table 2: Plasmids

Plasmid	Vector	Size (kbp)	Comment[a]	Reference
	pUC18	2.69	Amp^r, Rep_{Ec}, high-copy-number cloning vector	(6)
	pGEX-4T-2	4.97	Amp^r, Rep_{Ec}, expression plasmid	GE Healthcare
pILL600	pBR322	6.7	Amp^r, Km^r, Rep_{Ec}, source of Km resistance cassette (*aphA3´*-III)	(7)
pCJ450	pUC18	4.99	Amp^r, Rep_{Ec}, HP0540-HP0539 and flanking regions from *H. pylori* 26695A	This study
pCJ451	pUC18	6.18	Amp^r, Rep_{Ec}, pCJ450, HP0539 disrupted by *aphA3´*-III	This study
pCJ472	pUC18	4.98	Amp^r, Rep_{Ec}, pCJ450, SDM of RGD in HP0539	This study
pCJ474	pUC18	7.72	Amp^r, Km^r, Rep_{Ec}, pCJ450, HP0539 disrupted by *aphA3´*-III-*sacB*	This study
pCJ490	pUC18	4.97	Amp^r, Rep_{Ec}, pCJ450, SDM of LDQNT in HP0539	This study
pCJ491	pUC18	4.97	Amp^r, Rep_{Ec}, pCJ450, SDM of GISDK in HP0539	This study
pCJ492	pUC18	4.97	Amp^r, Rep_{Ec}, pCJ450, SDM of SEDVI in HP0539	This study
pCJ494	pUC18	4.97	Amp^r, Rep_{Ec}, pCJ450, SDM of NNEM in HP0539	This study
pCJ495	pUC18	4.97	Amp^r, Rep_{Ec}, pCJ450, SDM of FKTYM in HP0539	This study
pCJ496	pUC18	4.97	Amp^r, Rep_{Ec}, pCJ450, SDM of TSPSA in HP0539	This study
pCJ905	pGEX-4T-2	5.62	Amp^r, Rep_{Ec}, HP0539 expression plasmid, *H. pylori* 26695A *cagL* wild type	This study
pCJ1202	pUC18	4.97	Amp^r, Rep_{Ec}, pCJ450, SDM of ELFFI in HP0539	This study
pCJ1206	pUC18	4.97	Amp^r, Rep_{Ec}, pCJ450, SDM of AEQQC in HP0539	This study
pCJ1210	pGEX-4T-2	5.61	Amp^r, Rep_{Ec}, HP0539 expression plasmid, *H. pylori* 26695A *cagL* ΔRGD	This study
pCJ1234	pUC18	4.97	Amp^r, Rep_{Ec}, pCJ450, SDM of TASLI in HP0539	This study
pCJ1235	pUC18	4.96	Amp^r, Rep_{Ec}, pCJ472, SDM of RGD and TASLI in HP0539	This study
pCJ1236	pUC18	4.96	Amp^r, Rep_{Ec}, pCJ496, SDM of TSPSA and RGD in HP0539	This study
pCJ1237	pGEX-4T-2	5.59	Amp^r, Rep_{Ec}, HP0539/*cagL* expression plasmid, *H. pylori* 26695A *cagL* ΔRGD ΔTASLI	This study
pCJ1238	pGEX-4T-2	5.59	Amp^r, Rep_{Ec}, HP0539/*cagL* expression plasmid, *H. pylori* 26695A *cagL* ΔTSPSA ΔRGD	This study
pCJ1240	pUC18	4.99	Amp^r, Rep_{Ec}, pCJ450, SDM of T170K in HP0539	This study
pCJ1241	pGEX-4T-2	4.97	Amp^r, Rep_{Ec}, HP0539/*cagL* expression plasmid, *H. pylori* 26695A *cagL* ΔTASLI	This study
pCJ1242	pGEX-4T-2	5.62	Amp^r, Rep_{Ec}, HP0539/*cagL* expression plasmid, *H. pylori* 26695A *cagL* T170K	This study

[a] Amp^r, ampicillin resistance, Cm^r, chloramphenicol resistance, Km^r, kanamycin resistance, $Blast^r$, blasticidin resistance, Rep_{Ec}, replication origin for *E. coli*, Rep_{Hp}, replication origin for *H. pylori*

Supplementary Table 3: Primer pairs used for Site-Directed Mutagenesis (SDM) of HP0539/*cagL*

Method (SDM)	Primer name	Sequence (5´- 3´)	Deletion/Substitution
Inverse PCR	HP0539_aaDel_1_f	AATAATGAAATGGGTGAAGAAG	ΔTSPSA
	HP0539_aaDel_1_r	GGTTGAAAAAATCTCATCTAAG	
	HP0539_aaDel_2_f	GGTGAAGAAGATGCTCTAAACATC	ΔNNEM
	HP0539_aaDel_2_r	AGCACTAGGGCTAGTGGTTG	
	HP0539_aaDel_3_f	TTCAAAACTTATATGTCTAGCC	ΔSEDVI
	HP0539_aaDel_3_r	GATGAAAAATAACTCATTCG	
	HP0539_aaDel_4_f	TCTAGCCCTGAACTTTTATTAAC	ΔFKTYM
	HP0539_aaDel_4_r	AATCACATCTTCTGAGATGAAA	
	HP0539_aaDel_5_f	GTTTTAGTTCTTTATTGTGAAG	ΔGISDK
	HP0539_aaDel_5_r	GCATTGTTGCTCAGCAGTAT	
	HP0539_aaDel_7_f	GCTGAGCAACAATGCGGAATA	ΔLDQNT
	HP0539_aaDel_7_r	GGGATTGATTTTCATATAGGTTA	
QuikChange	HP0539_RGDdel_fw	CATCAAAAAAGCGGCCATTGCTTTGTTAGCGTTATT	ΔRGD
	HP0539_RGDdel_rv	CAAAATTGGCTTTCAATAACGCTAACAAAGCAATGG	
	ELFFI_Del_for	GCCAATTTTGAAGCGAATTCAGAAGATGTG	ΔELFFI
	ELFFI_Del_rev	CACATCTTCTGAATTCGCTTCAAAATTGGC	
	26695_AEQQC_Del_for	CCCTTAGACCAAAATACTGGAATATCCGAT	ΔAEQQC
	26695_AEQQC_Del_rev	ATCGGATATTCCAGTATTTTGGTCTAAGGG	
	26695_TASLI_Del_for	AGCAACATTGGAGGTACTGCTTCACAGACG	ΔTASLI
	26695_TASLI_Del_rev	CGTCTGTGAAGCAGTACCTCCAATGTTGCT	
	26695_TASLI_T170K_for	AGCAACATTGGAGGTAAAGCTTCCTTAATCACTGCTTCAC	T170K
	26695_TASLI_T170K_rev	GTGAAGCAGTGATTAAGGAAGCTTTACCTCCAATGTTGCT	

Supplementary Table 4: Primer pairs used for cloning, PCR and DNA sequencing

Primer name	Nucleotide sequence (5´- 3´)	Comment	Restriction site
Pcat-1	AACAGCTATGACCATGATTACG	Amplification of cm cassette	-
Pcat-2	AGAGGATCCGATATCGCATGCCTGCAGAG		*Bam*HI
Km1	CTGCTAAGGTATATAAGCTGGTGGG	Amplification of the km cassette	-
Km2	CATACTGTTCTTCCCCGATATCCTC		-
KanSacB_*Bgl*II_fw	TATAGATCTTCATGCTCTTTTAAATTTTGC	Amplification of the *aphA3´*-III-*sacB* cassette	*Bgl*II
KanSacB_*Bgl*II_rv	TATAGATCTCGAACCATTTGAGGTGATAG		*Bgl*II
HP0539_fw2	TATGAATTCCCATGGAAGATATAACAAGC	Cloning HP0539 into pGEX-4T-2	*Eco*RI
HP0539_rv2	ATAAGAATGCGGCCGCTCATTTAACAATGATCTTACTTG		*Not*I
HP0539_F1_II	ATATAGGATCCGATAAAGTTTTAGTTCTTTA	Deletion HP0539 (*cagL*::*aphA3´*-III)	*Bam*HI
HP0539_R1_II	ATATAGGATCCTCTTGGTAGGTGCTACC		*Bam*HI
pCAT-6	ATATGTGCAGGGCGTATTGCC	PCR and sequencing	
pCAT-8	TCCTGCAGATCTGTTGACG		
BssacB_F1	CTGCAAATCCCTGAACAGC	PCR and sequencing	
BssacB_R1	TCAAAGAGCTGTCTGATGC		
HP0539_seq_F	TGAATGGGATCAATGGAGAA	PCR and sequencing	
HP0539_seq_R	ATACTACAAATGCAAGTGAG		
pGEX4T_2F	TGGCAAGCCACGTTTGGTG	PCR and sequencing	
pGEX4T_2R	GTTTTCACCGTCATCACCG		
pUC/M13forward40	GTTTTCCCAGTCACGACG	PCR and sequencing	
pUC/M13rev	TCACACAGGAAACAGCTATGAC		

Supplementary References

(1) Nicholas K.B., Nicholas H.B. Jr., and Deerfield, D.W. II. 1997 GeneDoc: Analysis and Visualization of Genetic Variation, EMBNEW.NEWS 4:14.

(2) Biasini M., Bienert S., Waterhouse A., Arnold K., Studer G., Schmidt T. et al. SWISS-MODEL: modelling protein tertiary and quaternary structure using evolutionary information. Nucleic Acids Res. 2014; 42(Web Server issue):W252-8. doi: 10.1093/nar/gku340.

(3) Barden S., Lange S., Tegtmeyer N., Conradi J., Sewald N., Backert S., Niemann H.H. A helical RGD motif promoting cell adhesion: crystal structures of the *Helicobacter pylori* type IV secretion system pilus protein CagL. Structure 2013; 21(11):1931-41.

(4) Blum H., Beier H., Gross H.J. Improved silver staining of plant proteins, RNA and DNA in polyacrylamide gels Electrophoresis 1987; 8: 93-99.

(5) Yeo H.J., Yuan Q., Beck M.R., Baron C., Waksman G. Structural and functional characterization of the VirB5 protein from the type IV secretion system encoded by the conjugative plasmid pKM101. Proc. Natl Acad Sci USA 2003; 100, 15947-15952.

(6) Tomb J.F., White O., Kerlavage A.R., Clayton R.A., Sutton G.G., Fleischmann R.D. et al. The complete genome sequence of the gastric pathogen *Helicobacter pylori*. Nature 1997; 388(6642):539-547.

(7) Olbermann P., Josenhans C., Moodley Y., Uhr M., Stamer C., Vauterin M. et al. A global overview of the genetic and functional diversity in the *Helicobacter pylori cag* pathogenicity island. PLoS Genet 2010; 6(8):e1001069.

(8) Hanahan D. Studies on transformation of *Escherichia coli* with plasmids. J Mol Biol 1983; 166:557-580.

(9) Casadaban M., Cohen S.N. Analysis of gene control signals by DNA fusion and cloning in *E. coli*. J Mol Biol 1980; 138:179-207.

(10) Studier F.W., Moffatt B.A. Use of bacteriophage T7 RNA polymerase to direct selective high-level expression of cloned genes. J Mol Biol 1986; 189(1):113-130.

(11) Vieira J., Messing J.. The pUC plasmids, an M13mp7-derived system for insertion mutagenesis and sequencing with synthetic universal primers. Gene 1982; 19:259-268.

(12) Labigne-Roussel A., Courcoux P., Tompkins L. Gene disruption and replacement as a feasible approach for mutagenesis of *Campylobacter jejuni*. J Bacteriol 1988; 170:1704-1708.
